# The pathogenic mechanisms of *Tilletia horrida* as revealed by comparative and functional genomics

**DOI:** 10.1038/s41598-018-33752-w

**Published:** 2018-10-18

**Authors:** Aijun Wang, Linxiu Pang, Na Wang, Peng Ai, Desuo Yin, Shuangcheng Li, Qiming Deng, Jun Zhu, Yueyang Liang, Jianqing Zhu, Ping Li, Aiping Zheng

**Affiliations:** 1 Rice Research Institute of Sichuan Agricultural University, Wenjiang, Chengdu, Sichuan 611130 China; 2 Key laboratory of Sichuan Crop Major Disease, Sichuan Agricultural University, Wenjiang, Chengdu, Sichuan 611130 China; 3 Key Laboratory of Southwest Crop Gene Resource and Genetic Improvement of Ministry of Education, Sichuan Agricultural University, Yaan, Sichuan 611130 China; 4 Food Crop Research Institute, Hubei Academy of Agricultural Science, Wuhan, Hubei 611130 China

## Abstract

*Tilletia horrida* is a soil-borne, mononucleate basidiomycete fungus with a biotrophic lifestyle that causes rice kernel smut, a disease that is distributed throughout hybrid rice growing areas worldwide. Here we report on the high-quality genome sequence of *T. horrida*; it is composed of 23.2 Mb that encode 7,729 predicted genes and 6,973 genes supported by RNA-seq. The genome contains few repetitive elements that account for 8.45% of the total. Evolutionarily, *T. horrida* lies close to the *Ustilago* fungi, suggesting grass species as potential hosts, but co-linearity was not observed between *T. horrida* and the barley smut *Ustilago hordei*. Genes and functions relevant to pathogenicity were presumed. *T. horrida* possesses a smaller set of carbohydrate-active enzymes and secondary metabolites, which probably reflect the specific characteristics of its infection and biotrophic lifestyle. Genes that encode secreted proteins and enzymes of secondary metabolism, and genes that are represented in the pathogen-host interaction gene database genes, are highly expressed during early infection; this is consistent with their potential roles in pathogenicity. Furthermore, among the 131 candidate pathogen effectors identified according to their expression patterns and functionality, we validated two that trigger leaf cell death in *Nicotiana benthamiana*. In summary, we have revealed new molecular mechanisms involved in the evolution, biotrophy, and pathogenesis of *T. horrida*.

## Introduction

Rice is an economically important seed plant and the most important cereal food crop in the world; it provides approximately 20% of the world’s dietary energy supply^1,2^. Rice kernel smut (RKS), caused by the soil-borne Basidiomycete fungus *Tilletia horrida* that infects rice floral organs, was first reported in Japan in 1896^3^. RKS was once categorized as a minor disease with sporadic occurrence in rice-growing areas; however, the increasing demand for rice worldwide keeps driving the extensive planting of high-yielding cultivars and hybrid varieties. In China, the cultivated area of hybrid rice has reached about 1.6 million acres. In order to increase production, the breeding of three line hybrid rice that contains male sterile, maintainer, and restorer lines is undertaken. However, the rising incidence of exerted stigma in rice male sterile lines selected as part of the drive towards higher-yielding hybrid lines, increases the impact of RKS, which affects both the yield and quality of hybrid seed by producing masses of dark powdery teliospores^4,5^. This has led to an annual 40% to 60% prevalence of RKS in hybrid rice fields and to a 5–20% decrease in rice yield^6^. Disease incidence as high as 87% and 100% in hybrid rice fields has been reported in Pakistan and China, respectively^7^. RKS is now an increasing threat to rice cultivation in Asia, Oceania, Europe, America and Africa^8,9^.

Smuts are multicellular fungi, which exist as dark, thick-walled teliospores that are widespread in soil and the seeds of host plants. The morphological characters of 80 smut genera (4, 200 species) have been described and all species are parasitic on higher plants^10^. They infect many economically important hosts including maize, barley, wheat, rice, sugarcane, and forage grasses. The smut fungi have originated from two phylogenetically separate lines; *Tilletia* has different origins to *Ustilago* and *Sporisorium*^11^.

**Figure 1:**
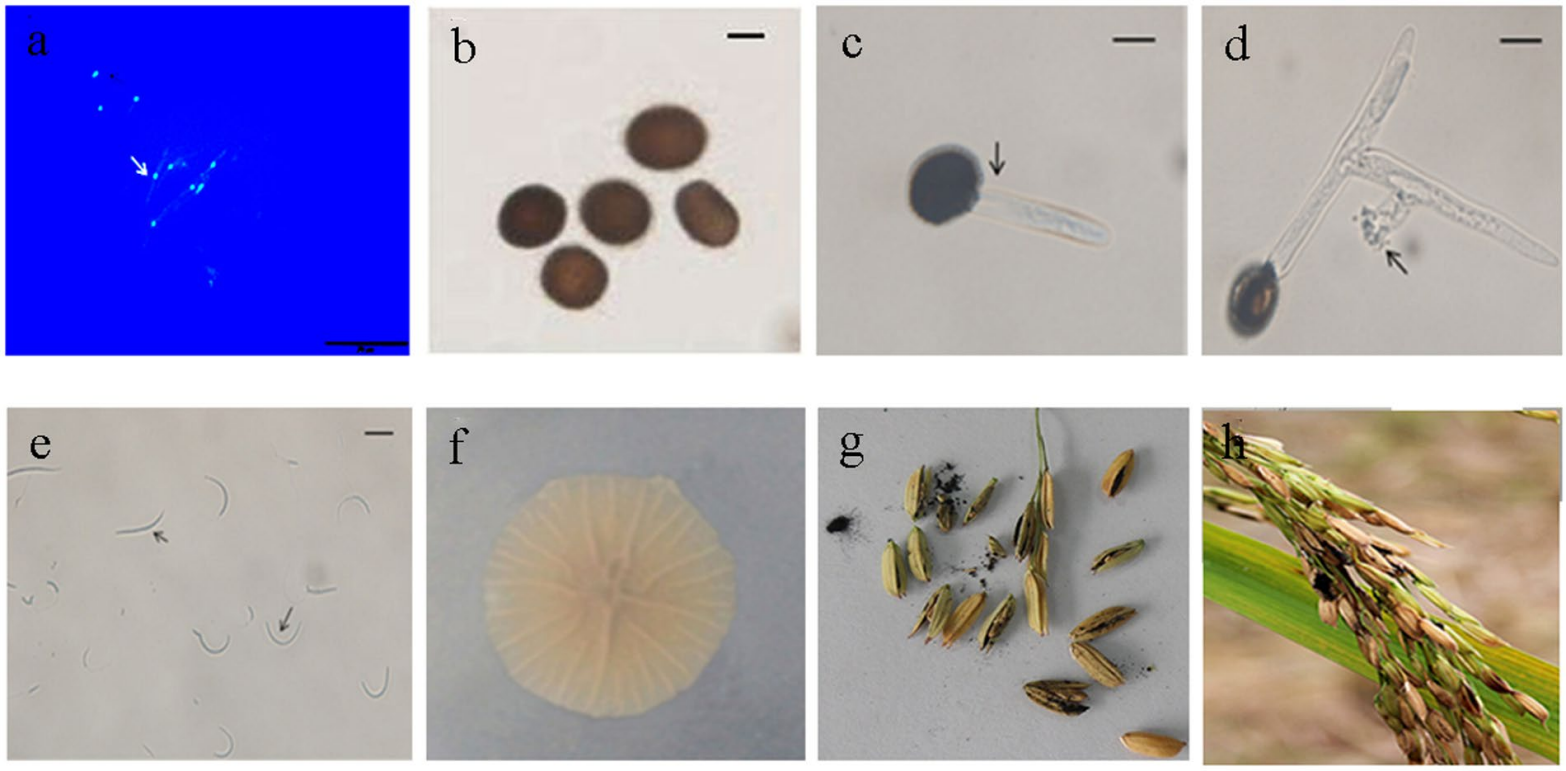
Characteristics of the *Tilletia horrida* strain. (**a**) A single nuclear mycelium stained with 4′, 6-diamidino-2-phenylindole (DAPI) after culture on PDA medium for 5 days, observed using a fluorescence microscope. Scale bars, 20 µm; (**b**) Teliospores under scanning microscopy; (**c**) and (**d**) Teliospores germination. (**e**) Morphology of secondary microspores. Scale bars, b-c: 10 µm; (**f**) Colony morphology of *T. horrida* after 15 d on PSA; (**g**) Hypha infection in rice kernels and growing points; (**h**) Kernel smut balls formed in rice spikelets.

*T. horrida* belongs to the *Tilletia* genus of the basidiomycota *Tilletiaceae* family. It possesses one nucleus per cell (Fig. 1a) and initiates infection through rice floral organs and the immature kernels, producing powdery dark teliospore balls in the rice kernels during the late phase of infection. Teliospore balls are black and spherical, measuring 25_˜_30 × 23_˜_30 *µ*m, and possess colorless indentations on their surface (Fig. 1b). The spores can survive for more than 1 year in the soil, and for more than 3 years in rice seed that has been infected at the flower stage^5^. On germination, the teliospores produce a promycelium (Fig. 1c) that display distal verticillated digitations (Fig. 1d). Microspores grow in these verticillated digitations and the appearance of these secondary microspores is linear or curved (Fig. 1e,f). The early infective stage of *T. horrida*, remains asymptomatic, and the organism grows systemically until masses of dark powdery spores appear on the grains (Fig. 1g,h). Besides infecting *Oryza sativa*, *T. horrida* is known to infect wild rice and certain weeds, and this may aid the spread of inocula to healthy rice plants at the flowering stage. Because *T. horrida* fungi are biotrophic pathogens, their growth on artificial media is slow. Studies on this species have predominantly focused on morphological characteristics and its evolutionary biology. To date, genomic structure and pathogenic mechanisms of *T. horrida* have not been studied.

Despite the high rice yield losses worldwide caused by *T. horrida*, there has been a reliance on chemical fungicides to control RKS^12^. To data, no investigations have been carried out to identify cultivars that are completely immune to *T. horrida*. In order to exploit more effective resistant cultivars, a better understanding of the pathogenic mechanisms of *T. horrida* is needed. In the current study, we examined the possible molecular basis of host-pathogen interactions and the pathogenic mechanisms of *T. horrida* through the sequencing, assembly, and annotation of the genome. The up-regulation of candidate effector genes encoding secreted proteins suggests a list of candidate virulence factors that may play important roles in pathogenicity. A comparative genomics study with the four smut fungi related to *T. horrida*, namely *Sporisorium reilianum*, *Sporisorium scitamineum*, *Ustilago maydis*, and *Ustilago hordei* has also been conducted, which may provide useful insights for improving rice crop yields. In summary, this work has elucidated the interactions between rice and the fungal pathogen, *T. horrida*.

## Results

### Genome sequencing and assembly

The genome of the *T. horrida* strain JY-521 was sequenced using a PacBio RS II sequencing strategy and three single-molecule real-time (SMRT) sequence cells were obtained (Supplementary Table S1). The three SMRT cells were assembled into scaffolds using MinHasf Alignment Process (MHAP) assemble, resulting in longer sequences with a total assembly size of 23.2 Mbp; 84 contigs and 84 scaffolds (no gaps within them) were assembled. The N50 size of the scaffold and contigs was 538,348 bp. The average length of the scaffold and contigs was 276,373.48 bp, and the maximum length was 1,812,755 bp. The mitochondrial genome of *T. horrida* was assembled as a circular molecule of 98.96 kbp, and the GC content of the genome was 55.67% (Table 1, Supplementary Table S2). The genome assembly statistics of *T. horrida* and other smut fungal isolates, such as *U. hordei*, *S. scitamineum*, *S. reilianum*, and *U. maydis*, are shown in Table 1. The results revealed that the genome size of smut species was small, and the size of *T. horrida* JY-521 was greatest of the five smut fungi tested. In the genome of *T. horrida*, the average gene length and introns per gene were 2,085.6 bp and 4.14, respectively, these were the largest of the five fungi genomes (Table 1).

**Table 1:** Genome characteristics of five smut fungi.

Features	*Tilletia horrida*	*Ustilago hordei*	*Sporisorium scitamineum*	*Sporisorium reilianum*		*Ustilago maydis*
Host	Rice	Barley	Sugarcane	Sorghum		Maize
Size (Mbp)	23.2 Mb	21.15 Mb	19.42 Mb		18.38 Mb	19.66 Mb
%G + C content	55.67	52.16	55.16		59.87	54.03
% Repeat	8.45	4.69	1.58		2.34	4.32
Protein-code genes	7,729	7,110	7,711		6,648	6,783
Average gene length (bp)	2,095.6	1,782.49	1,568.52		1,853.55	1,800.18
Exons sequence(bp)	13,596,636	12,126,273	12,264,531		11,923,380	12,019,288
tRNA	155	—	—		—	111
Gene density (# gene per Mbp)	332.86	336.17	397.06		361.5	345.02
Average intron length (bp)	678.66	410.00	435.00		327.51	366.82
Introns per gene	4.14	1.54	1.7		1.45	1.43
Average exon length (bp)	424.69	1107.3	861.66		1232.76	1238.58

Statistics are presented relative to genes, coding exons, introns and intergenic regions (sequences between two adjacent genes) in the *T. horrida* genome. Comparisons between *T. horrida* and alternative smut fungi were restricted to *T. horrida* regions that aligned to the alternative smut sequences across their full length.

### Genome annotation

A total of 7,729 protein-coding genes were annotated, with an average size per gene of 1,759.17 bp with 6,973 being supported by the RNA-seq data. Most of the coding sequences (CDSs) were between 500 and 2,000 bp in length (4,667) and only a few were larger than 5,000 bp (Supplementary Fig. S1). This coding DNA contained 13,596,636 bp of exon sequence lengths (Table 1). Of these 4,960 (64.17%), 2,995(38.75%), 5541(71.69%), 4,324 (55.95%) and 6,212(80.37%) genes were predicted to have homologies with known functions in the SwissProt, Gene Ontology (GO), Kyoto Encyclopaedia of Genes and Genomes (KEGG), EuKaryotic Orthologous Groups (KOG), and Non-Redundant Proteins (NR) databases, respectively (Supplementary Table S3). With regard to non-coding genes, we identified 155 transfer RNAs, and 41 ribosomal RNA fragments from the assembly (Table 1, Supplementary Table S4).

### *T. horrida* repeat sequence content

**Figure 2:**
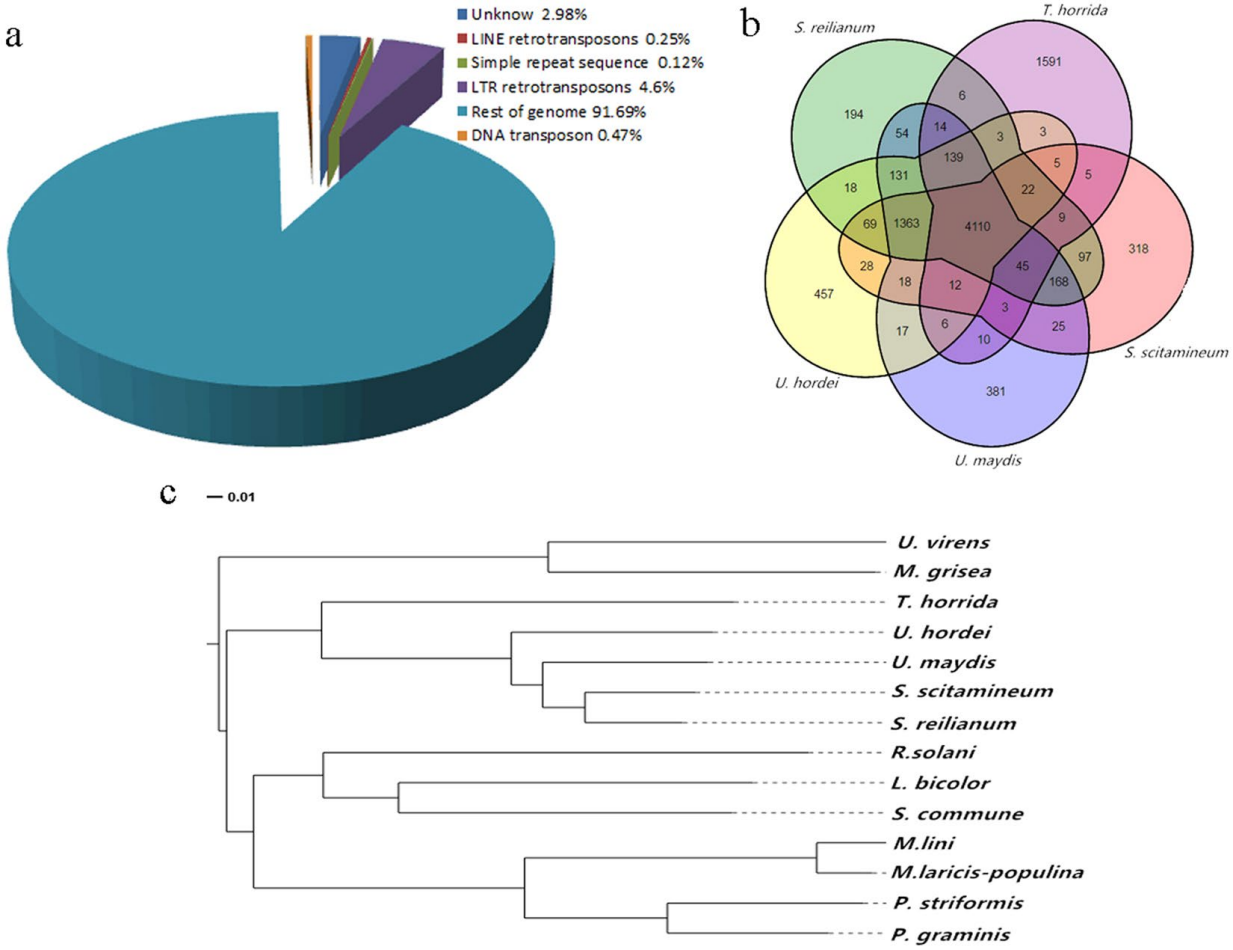
Phylogenetic relationship of *Tilletia horrida* with other Basidiomycetes fungi. (**a**) The proportion (%) of different types of repetitive sequences in the *T. horrida* JY-521 genome. LINE: long interspersed elements, LTR: long terminal repeat retrotransposons. (**b**) Venn diagram showing orthologs between the five sequenced smut fungi. The values explain the counts of ortholog groups and the counts of genes in parentheses. (**c**) The phylogeny of 12 Basidiomycota fungi and 2 Ascomycota fungi. The phylogeny was constructed using Mega 6 with 806 single-copy gene. Protein alignments were analyzed using MUSCLE3.8.31.

Repeat DNA sequences comprise interspersed repetitive and tandem repeats that are an important part of a genome. Tandem repeats comprise microsatellite sequences and minisatellite sequences. Interspersed repetitive sequences, also known as transposable elements (TEs), comprise DNA transposon, long terminal repeat (LTR) reverse elements, and long interspersed repetitive elements. Transposable elements that play an important role in fungal pathogens are predominant in repeat sequences^13^. The *T. horrida* genome is comprised of 1,958,189 bp DNA transposons and retrotransposons that include 76 families. They accounted for 8.3% of the 23,215,372 bp genome sequences. TEs represented about 64.1% of the repetitive sequences and constituted the majority of these sequences. There were 86.4% LTR retrotransposons in TEs (Fig. 2a, Supplementary Table S5). Among the repetitive elements, gypsy elements comprised 835,814 bp, and accounted for 43.36% of the TEs and 3.6% of the total assembly (Supplementary Table S5). These were the most abundant type of TEs and we compared the repeat sequences of the five smut fungal species genomes; results showed that the ratio of repeat sequences in the *T. horrida* genome was highest of the five fungi tested (Table 1). This indicated that repetitive sequences may play a more important role in *T. horrida* during inoculation.

### Evolution and comparative genomics

For comparative genomics, six additional RKS strains collected from different geological regions in China (Supplementary Table S6) were subjected to 50× sequencing. A comparison was made of the number of single nucleotide polymorphisms (SNPs) between the JY-521 genome and the six other strains (Supplementary Table S7). The smallest number of SNPs was 69,953 when JY-521 was compared with GZ-102 (collected from Guizhou Province) and the greatest number of SNPs was 124,367 when JY-521 was compared with HN-145 (collected from Hunan Province); these findings demonstrated a low level of sequence variation among the different *T. horrida* strains. The low nucleotide diversity in the genotypes showed a low level of intraspecific sequence variation, suggesting that variations between *T. horrida* populations were not obvious.

The phylogenetic tree of *T. horrida* and 13 other fungal species (11 Basidiomycota and 2 Ascomycota outgroups) was evaluated using a set of highly conserved single-copy genes. The analysis revealed that *T. horrida* was more closely related to the other four smut fungi than to other species, including the rice pathogen *Rhizoctonia solani* and the mushroom fungus *Laccaris bicolor* (Fig. 2c). Furthermore, the five smut fungi *T. horrida*, *U. hordei*, *S. reilianum*, *S. scitamineum*, and *U. maydis* had fewer genes than other fungi studied (Supplementary Table S8). The *T. horrida* genome consisted of 7,729 genes, and of these genes, 1,991 were unique; there were 5,983 families, and of these 1,089 were single-gene families. *T. horrida* possessed the greatest number of genes among the five smut fungi (Supplementary Table S8).

Synteny analysis between *T. horrida* and *U. hordei* found no obvious co-linearity (Supplementary Fig. S2a); however, *U. hordei* and *U. maydis* was predominantly co-linear (Supplementary Fig. S2b). This suggests that *Tilletia* belongs to a phylogenetically separate clade when compared with *Ustilago* and *Sporisorium*. A total of 4,410 gene families were shared among all five smut fungi, and there were 2,472 predicted genes in 1,591 gene families that appeared to be unique to *T. horrida* (Fig. 2b). Among these unique genes, genes of unknown function accounted for 59.66%, 138 genes belonged to unnamed or uncharacterized protein-encoding genes, and the expression of 62 genes was up-regulated during early infection (Supplementary Fig. S3). These unique genes may play important roles in pathogenicity that need to be explored. Gene families that had a large number of members included ATP-binding cassette (ABC) transporter, TPR-like protein, NAD (P)-binding protein, amino acid transporter and the glycoside hydrolase family.

Phylogenetic analysis also demonstrated a high degree of homology between *T. horrida* and *U. hordei*, *U. maydis*, *S. scitamineum*, *S. reilianum*, but this was displayed in different branches; *T. horrida* and *U. hordei* showed a closer relationship than *S. scitamineum*, *S. reilianum*, and *U. maydis*. The results provided an interpretation of the evolution of living smut fungi and the diversity of their hosts. The four related smut organisms could be used as reference species to map the *T. horrida* genome. Moreover, the fact that *T. horrida*, *U. maydis*, *U. hordei*, *S. scitamineum* and *S. reilianum* share most gene families in common supports the evolutionary relationship between these species (Fig. 2b).

### Transcriptome analysis during infection

To detect the key disease-associated genes expressed during the entire infection process, we analyzed the transcriptomes of *T. horrida* at six time points. We found that 6,973 genes were expressed during the entire infection process. As compared with axenic cultures, there were 120, 62, 29, 64, and 32 genes significantly up-regulated, and 217, 49, 62, 25, and 23 genes were significantly down-regulated at 8, 12, 24, 48, and 72 h post inoculation, respectively (FDR < 0.05 and |log_2_ Fold Change| > 1; Supplementary Fig. S4a,b). We analyzed gene expression relation at six stages of infection, and found that the gene expression pattern at 8 h was similar to 12 h post inoculation, while 48 h and 72 h were similar (Supplementary Fig. S5). It was clear that the pattern of gene expression was altered following infection and that the expression of pathogenicity-related genes was associated with the stage of infection.

**Figure 3:**
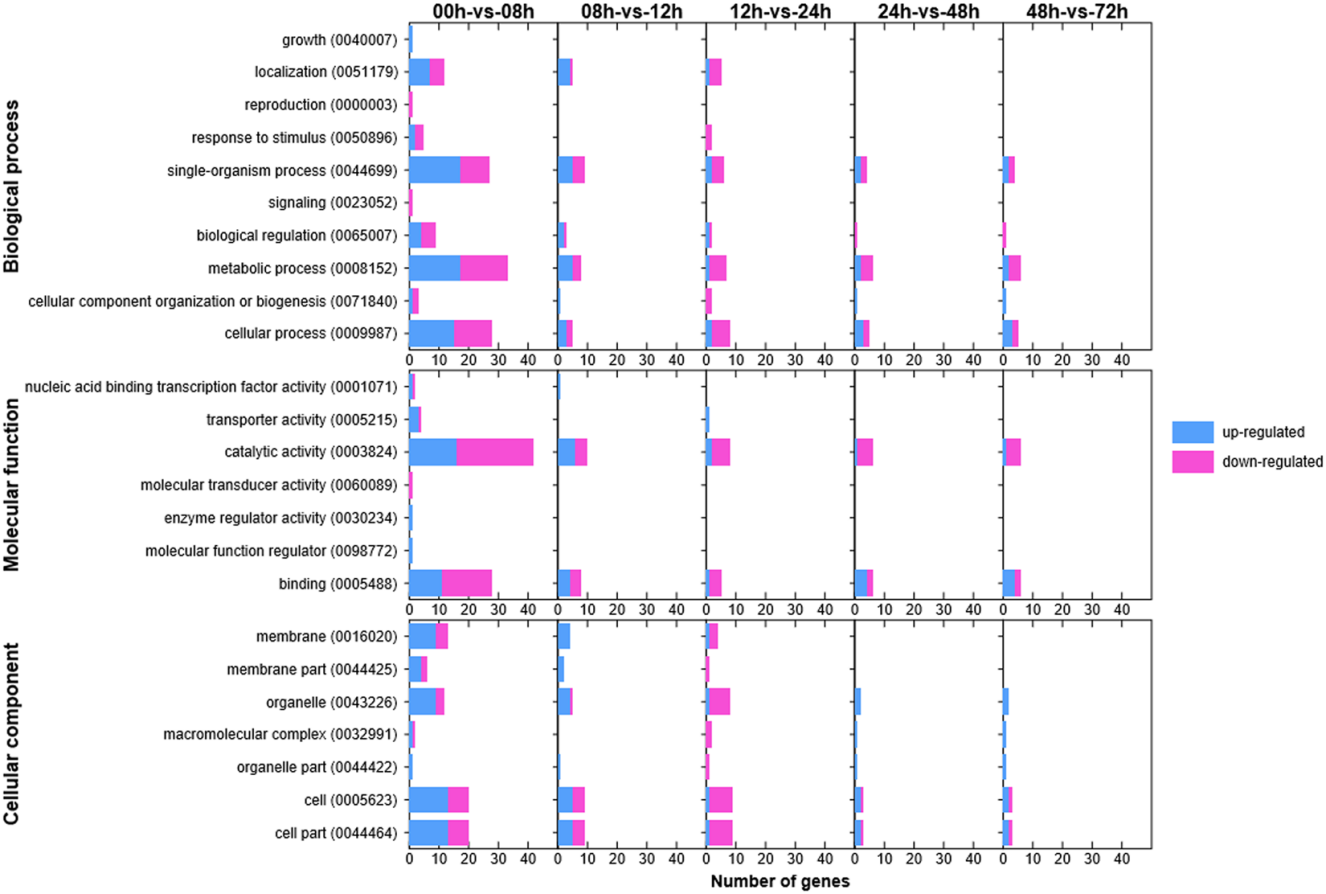
Up-regulated and down-regulated genes according to Gene Ontology (GO) annotation.

The numbers of up-regulated genes within different GO categories and terms at different stages of infection were compared (Fig. 3). The three GO categories included “biological process”, “cellular component”, and “molecular function”. GO enrichment analyses revealed correlations between stage of infection and gene expression for a range of GO terms within these categories. The up-regulated genes for the following GO terms had high numbers during the host infection process: GO:0044699 single-organism process, GO:0008152 metabolic process, and GO:0009987 cellular process in biological processes, GO:0003824 catalytic activity, GO:0005488 binding in molecular components, GO:0016020 membrane, GO:0043226 organelle, GO:0005623 cell, and GO:0044464 cell part in cellular functions. Some uniquely enriched genes within the GO classes showed the same expression tendency. Although the direct relationship of these genes to pathogenesis was not confirmed, it was possible to use the transcriptome pattern information to demonstrate trends.

### Genes involved in pathogenicity

**Figure 4:**
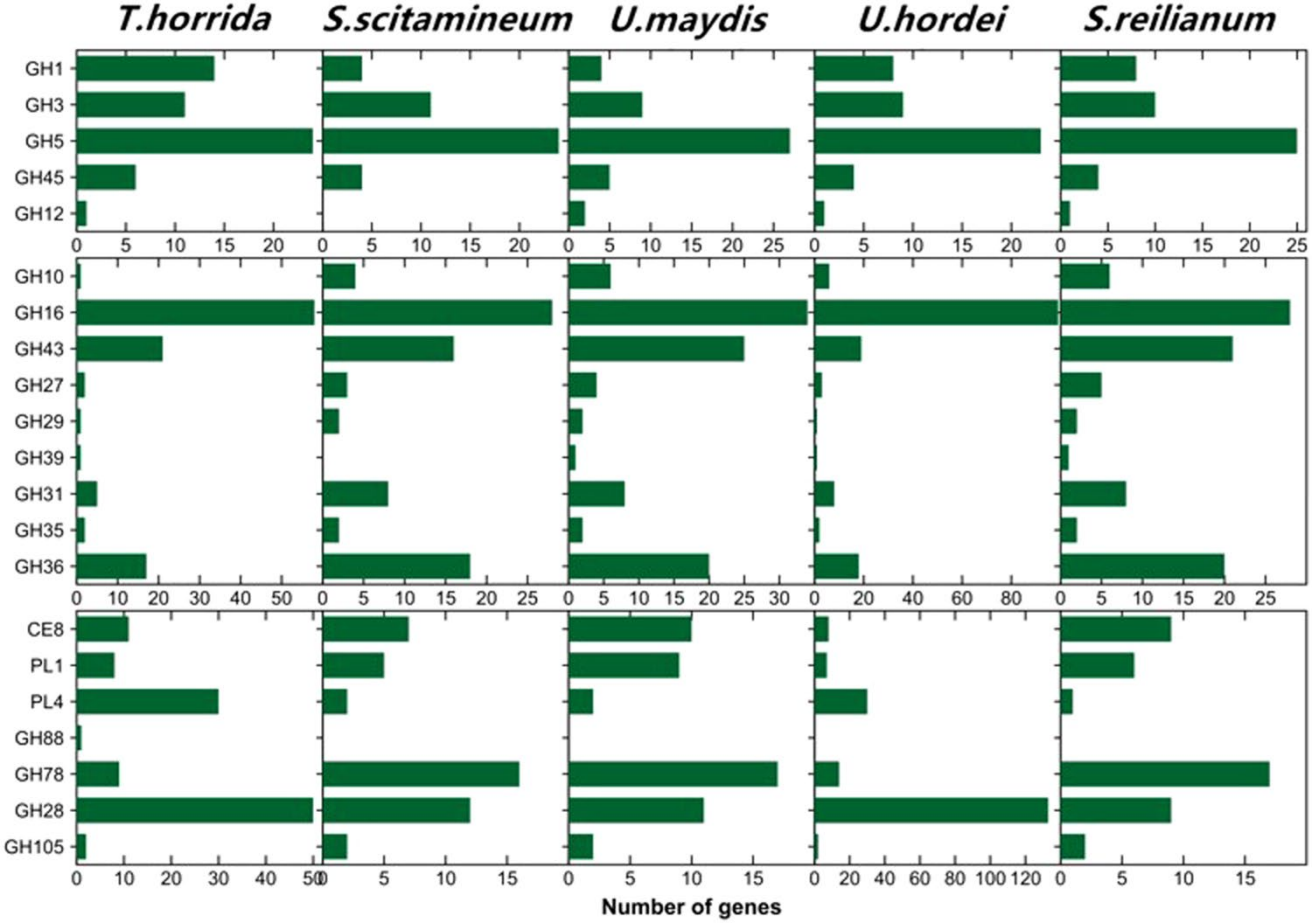
Summary of *Tilletia horrida* genes assigned with CAZyme functional annotations.

To successfully degrade plant cell walls and infect a host plant, phytopathogenic fungi secrete carbohydrate-active enzymes (CAZymes) that include glycoside hydrolases (GH), glycosyl transferases (GT), polysaccharide lyases (PL), and cutinases^14^. We predicted 1,424 putative CAZymes genes in the *T. horrida* genome; this is similar to biotrophic pathogenic fungi, such as *U.virens*; but it is significantly lower than plant hemi-biotrophic fungi, such as *M. grisea* (Supplementary Table S9, Fig. 3). The putative CAZyme genes comprised 542 families, including 474 GH, 533 GT, 101 carbohydrate esterases (CE), 273 carbohydrate binding modules (CBM) and 43 PL (Supplementary Table S9). Of these, the GH and CBM families were reduced in comparison with *M. grisea*, particularly the proteins in such families as GH6, GH10, GH74, GH39 and GH43 that are involved in degrading the cellulose, hemi-cellulose, and pectin of plant cell walls (Supplementary Dataset 1)^15–17^. Among the five smut fungi, the expression levels of CAZyme genes showed a similar trend. However, *T. horrida* had some conspicuously enriched PL4 (involved in pectin degradation) families (Fig. 4). We found that the number of CAZyme genes differentially expressed in *T. horrida* was similar to *U. hordei*; this may be because the rice host of *T. horrida* is closely related to the barley host of *U. hordei*. These analyses suggest that they had common polysaccharide degradation machinery.

**Figure 5:**
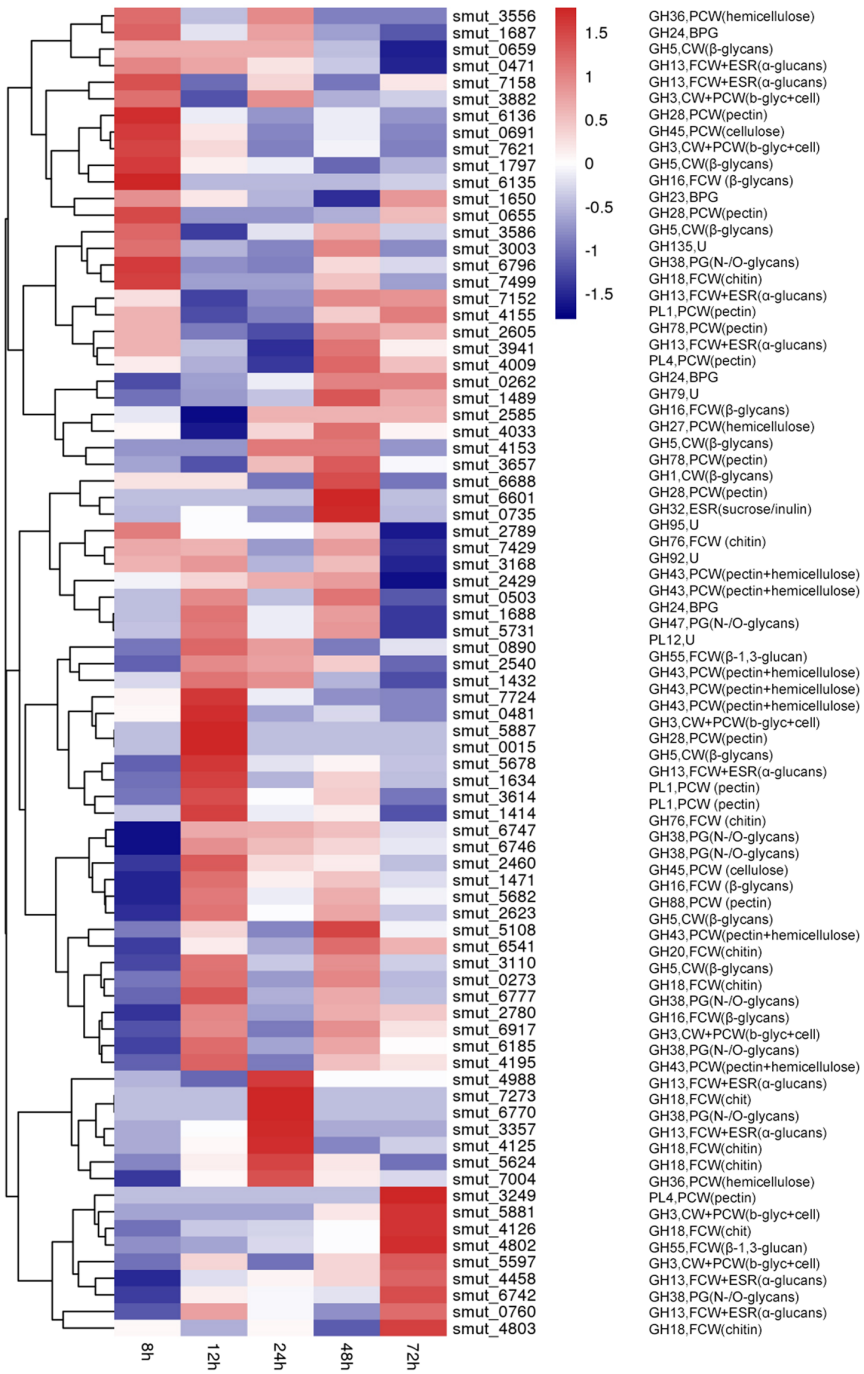
The expression patterns of genes coding carbohydrate degradative enzymes of *Tilletia horrida*.

A putative analysis was undertaken of the expression of genes encoding CAZymes over the six infection stages. There were 960 genes that showed specific transcript expression patterns at different infection stages. Finally, 652, 912, 542, 594, and 185 genes encoding CAZymes were upregulated at 8–72 h after infection (Supplementary Table S10). Genes encoding GH family members involved in the infection process reached maximum expression at 12 h. Genes for cellulose-degrading enzymes and hemicellulose-degrading enzymes reached maximum expression at 12 h, while the genes for pectin-degrading enzymes reached maximum expression at 12 h and 48 h, respectively (Supplementary Table S11). These results explain the degeneration of plant primary cell walls and the middle secondary walls during early infection. Genes of the CAZyme families were differentially expressed during the six stages, though not in a consistent fashion. GH16, GH5, GH43, GH28, GH36, CE8, and PL1 showed the highest expression levels, which suggests that the encoded enzymes were present at higher activity during infection and are associated with pathogenicity (Fig. 5 and Supplementary Table S11). Different gene expression patterns clearly occurred at different infection times, and it appears that the expression of genes encoding degradation-associated enzymes plays an important role during infection.

As a biotrophic pathogenic fungus, *T. horrida* is expected to possess some pathogen-host interaction (PHI) genes^18^. A total of 1,697 putative PHI genes were identified in the *T. horrida* genome. Transcript analysis was performed to identify differential expression patterns of these 1,697 genes during the six infection stages (0, 8, 12, 24, 48, and 72 h). These results suggest that 1,289 genes were expressed at all stages and mainly encoded cell wall degrading enzymes, proteins related to energy metabolism, and proteins related to membrane transport. Of them, 71 were significantly down-regulated and 64 significantly up-regulated genes after infection; the expression pattern of these genes is shown in Supplementary Fig. S6. The expression of *smut_3036*, which encodes a protein putatively associated with a toxin, reached a maximum at 12 h. The gene *smut_4052* encodes carbohydrate esterase Family 5 proteins that are associated with cell wall degradation. The activity of this protein is probably an important determinant of the virulence of pathogenesis. The functions of these virulence associated genes could be clarified by gene disruption or complementation studies.

Plant pathogenic fungi produce some secondary metabolites that are related to pathogenicity, such as host-selective toxins^19^. In total, 77 putative secondary metabolism-related genes and seven gene clusters were identified in the *T. horrida* genome (see Supplementary Fig. S7); the seven gene clusters comprised three polyketide synthase (PKS) clusters, three nonribosomal peptide synthetase (NRPS) clusters, and one prenyltransferase hybrid cluster; the core gene of every cluster is shown in Supplementary Table S12. These genes are important in the biosynthesis of various secondary metabolites^20,21^. Iron is an important element necessary for many essential processes in living organisms. *T. horrida* acquires iron from host rice cells by synthesizing iron-chelating siderophores. Three NRPS gene clusters may participate in siderophores production; they include gene encoding ferrichrome peptide synthetase (*Smut_0071*), 4-coumarate-CoA ligase (*Smut_0795*), and polyketide synthase (*Smut_1014*) and their expression was up-regulated at 24 h after infection (Supplementary Fig. S7). We identified the 12,639 bp core gene *Smut_0071*, which participates in the synthesis of ferrichrome siderophore peptide^22^. In the *S. scitamineum* genome, *SmutADNA4_GLEAN_10002728*, which encodes ferrichrome siderophore peptide, has 75% identity with *Smut_0071*.

Cytochrome P450s (CYPs) play an important role in pathogenesis and in the production of toxins^23,24^. Transporters are not only involved in obtaining carbon sources and nitrogen sources from their host plants, but are also involved in toxin and effector secretion^25^. In total, 19 CYP genes were identified in the *T. horrida* genome and their expression is shown in Supplementary Fig. S8. In addition, we also found 254 transporter genes in the *T. horrida* genome, among them comprise 40 ATP-binding cassette (ABC) superfamily transporters genes. Therefore, despite the presence of fewer metabolites, it could be secreted extracellularly.

### The *T. horrida* secretome

**Figure 6:**
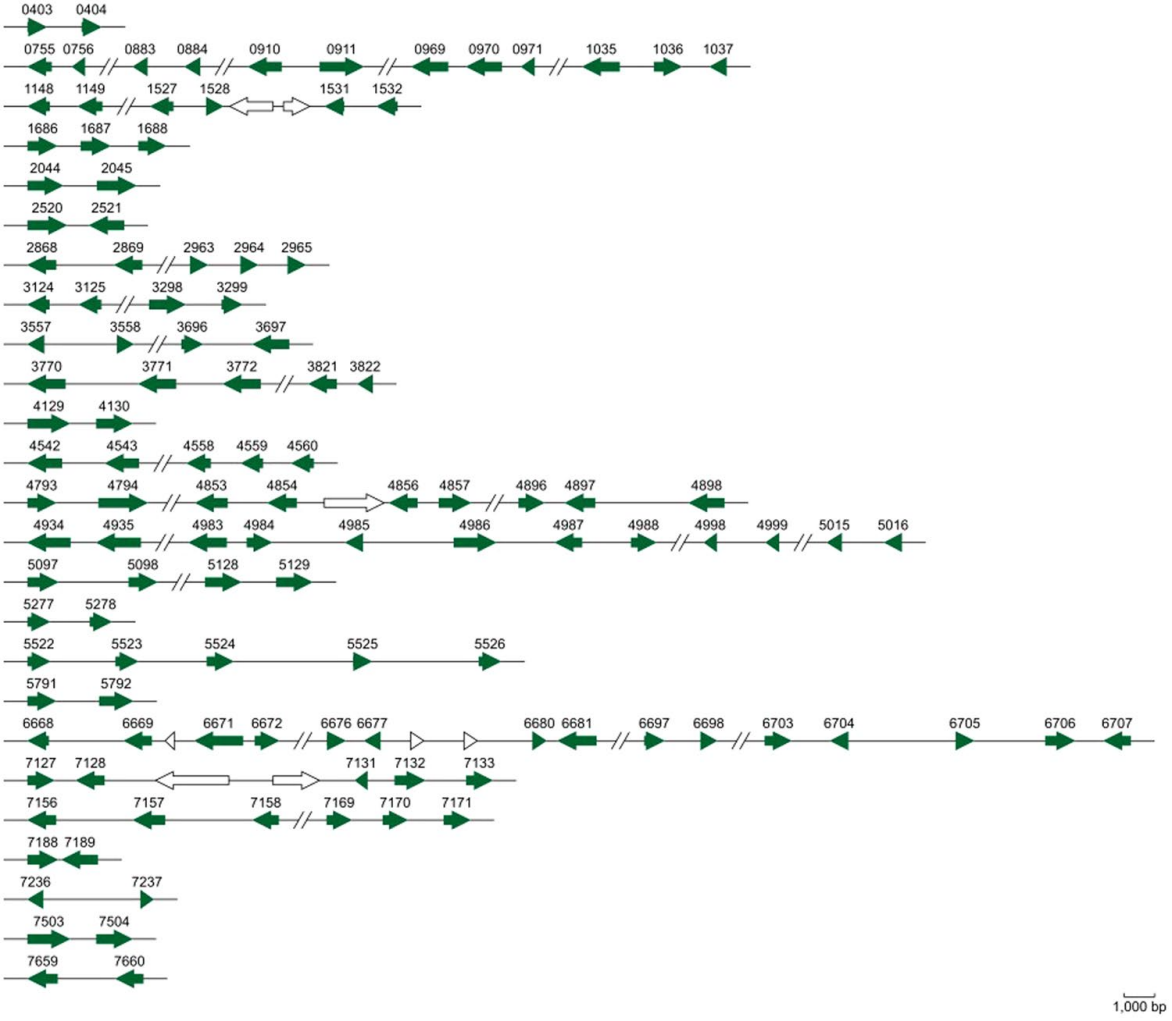
Genes and gene clusters encoding putative effectors in the *Tilletia horrida* genome. 120 putative effector genes are aggregated into 50 clusters. Shaded arrows represent putative effector genes while white arrows denote other genes.

**Figure 7:**
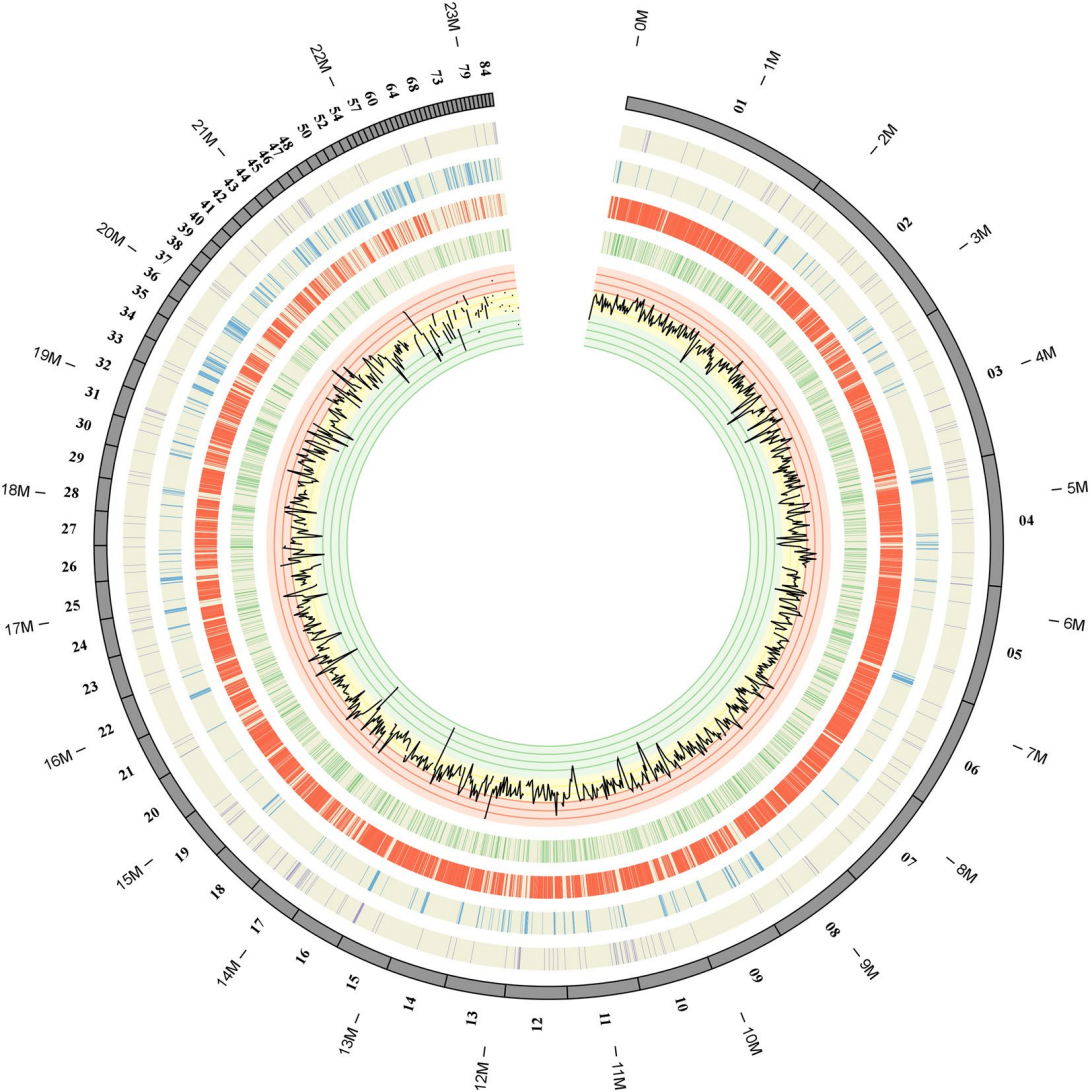
Physical locations of predicted secreted protein genes, all protein-coding genes and PHI-base genes in relation to regions of repetitive sequences and GC content distribution in the assembled genome of *T. horrida*. From outside to in: Location of genes encoding predicted secreted proteins (purple) in the assembled genome; The distribution of transposable elements (blue) in the *T. horrida* genome; Single-copy DNA regions (red) of the *T. horrida* genome; Locations of the PHI-base gene homologues (green) involved in pathogen–host interactions; Graphs of GC (black) contents. Areas of low GC correspond well to regions of repetitive DNA. The maps were drawn with OmniMapFree.

Recent research has revealed that the secretion of proteins from pathogens is related to the progression of an infection, especially for biotrophic fungi that have intimate host-fungal interactions^26^. A total of 597 potentially secreted proteins (7.72% of the proteome) of *T. horrida* were predicted (Supplementary Table S13)^27,28^. *T. horrida* had a higher secretome than *S. reilianum*, but lower than *U. hordei, U. maydis*, and *S. scitamineum* (Supplementary Table S13). There were 366 genes encoding small (<400 amino acids) secreted proteins (Supplementary Fig. S9), and of these, 131 genes were upregulated at 8 h after infection (Supplementary Fig. S10); these were identified as potential plant effectors. In the maize pathogen *U. maydis*, it was observed that most of the genes in secreted protein-coding gene clusters were induced simultaneously in infected tissue^29^. For *U. maydis*, there were 120 predicted effector genes organized into clusters, suggesting that local duplications might be involved in the expansion of effectors in *T. horrida* (Fig. 6). Repetitive elements are known to have been crucial factors throughout genome evolution and in the diversification of functional genes. However, a relationship between candidate effectors and TE-rich regions (low GC content) was not demonstrated in the current study (Fig. 7). This suggests that TE-driven evolution might have only a small influence on the interactions of *T. horrida* and rice.

### *T. horrida* candidate effectors and their validation

Some plant pathogen effectors could impact host cells through their various functions; furthermore, the receptors of pathogen effectors operate as dominant disease susceptibility genes^30^. The effector proteins from rice sheath blight pathogen and rice blast fungus have previously been studied^31,32^; however, no effector of the RKS pathogen has been reported prior to the current research.

**Figure 8:**
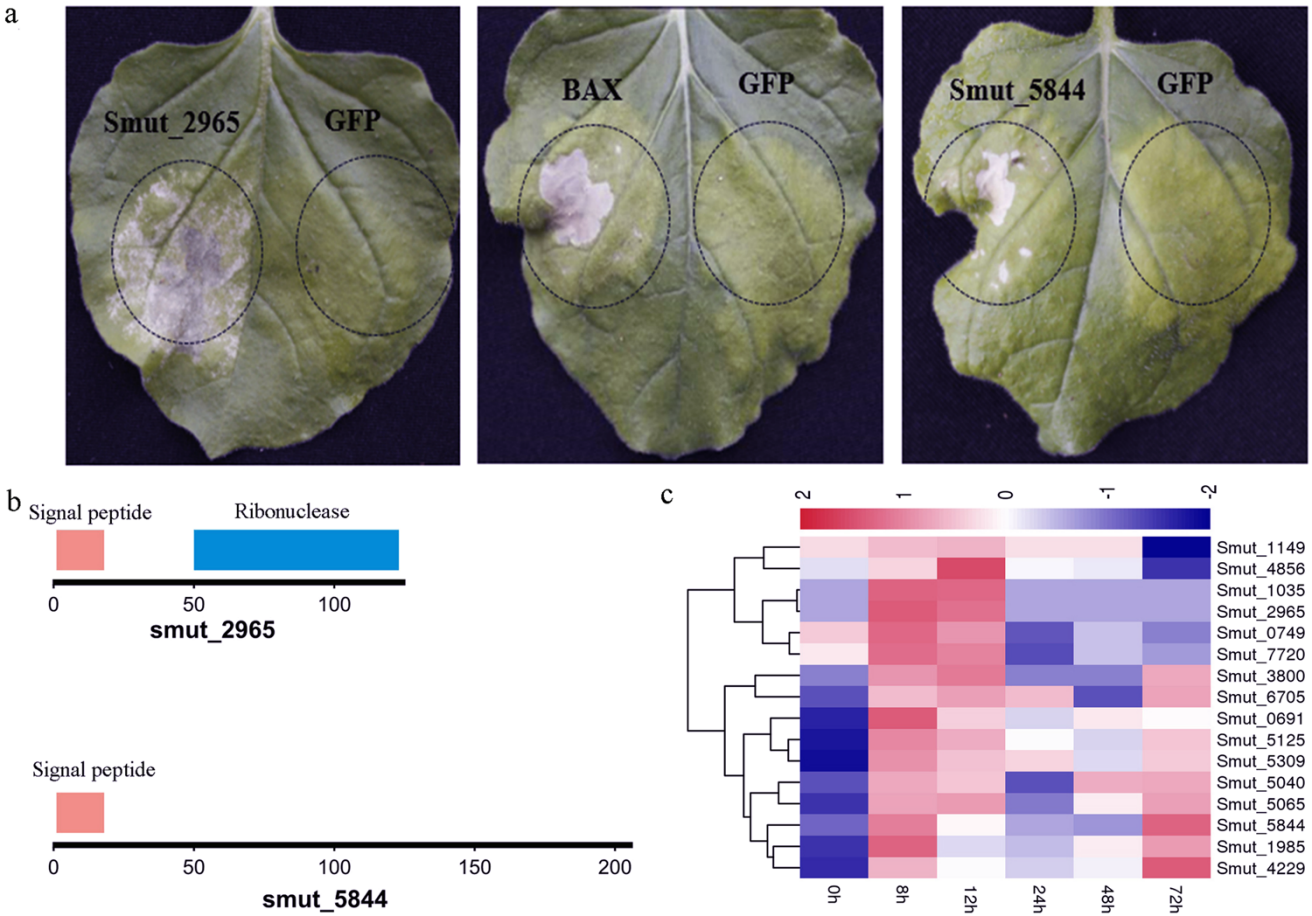
Candidate effectors cause cell-death in *Nicotiana benthamiana*. (**a**) Phenotypes observed on *N. benthamiana*. The effectors are encoded by the *smut_2965* and *smut_5844* genes, respectively, and the cell death phenotypes were visible 4 d after inoculation with purified proteins. BAX gene was used as a positive control, and the GFP as the negative control. (**b**) Genes with signal peptides (red) and domain structures. Ribonuclease domains (blue) are identified from the Pfam database. (**c**) Clustering of predicted effectors.

To identify novel potential effectors, we selected 131 candidate genes from the 366 genes that encoded secreted proteins and demonstrated upregulated expression during early infection (Supplementary Fig. S10). The potential effector gene expression vectors were transformed into *Nicotiana benthamiana* using the *Agrobacterium*-mediated transformation method. Interestingly, genes for two potential secreted effectors, *smut_2965* (ribonuclease domain) and *smut_5844* caused cell death of *N. benthamiana* leaf phenotypes after inoculation at 4 d (Supplementary Fig. S11, Fig. 8a,b). The highest level of infection was expressed at 8 h after infection. Furthermore, it is known that protein effector genes from pathogens undergo rapid duplication, diversification, deletions, and mutations^33^. We suggest that some proteins active in host cells are delivered by the fungus to trigger a defense response; however, this requires further investigation.

Additionally, in order to determine other potential effectors, 78 significantly up-regulated expressed secreted protein genes (FDR < 0.05 and |log2 Fold Change| > 1) in *T. horrida* were selected and classified into 16 clustered profiles based on trends observed in gene expression using Short Time-series Expression Miner software (STEM) (Supplementary Fig. S12). The clustered profiles with p ≤ 0.05 were considered as statistically significant. In Supplementary Fig. S12, profiles 18 and 19 show two gene clusters that have the same expression trend with two verified effectors, respectively. In them, there are 14 secreted protein genes as potential effectors (Supplementary Table S14) and their expression patterns recorded at different infection times are shown in Fig. 8c. Interestingly, there are three genes, namely *smut_1035*, *smut_1149*, and *smut_4856* organized into clusters with *smut_2965* in the *T. horrida* genome (Fig. 6). We speculated that those genes were involved in the regulation of pathogen-host interaction. We also analyzed the homologous genes of verified effectors in five smut pathogens; results showed that there were many effector genes homologous with *smut_2965* (Supplementary Table S15), but not with *smut_5844*. We suspect that *smut_5844* is a gene for a new effector protein that only affects rice. Of the two *T. horrida* effectors, we observed fewer SNP among the six RKS strains (Supplementary Dataset 2), suggesting a low intraspecific genetic diversity among the *T. horrida* population, even with regard to the effectors.

### Novel virulence-associated factors in the transduction signal pathway

In order to infect the host plant, plant pathogenic fungi must make appropriate responses to a variety of host-plant surface environmental receptors. These receptors function via the mitogen-activated protein kinase (MAPK) pathway that communicates a signal from a receptor on the surface of the cell to the nucleus in the cell. G protein-coupled receptors (GPCRs) and G-proteins are important components of the MAPK pathway, which has seven transmembrane domain receptors, constitutes a large family of cell surface receptors and is responsible for transducing extracellular signals into intracellular responses that involve complex intracellular-signaling networks^34,35^. GPCRs, which are extremely diverse in sequence and function, are found only in eukaryotes, including yeast, choanoflagellates and animals^36^.

Here, we identified GPCR-like proteins in 14 fungi. *T. horrida* had 67 GPCRs, which were grouped into 22 families (Supplementary Dataset 3). Therefore, the number of GPCRs in *T. horrida* was smilar to *U. hordei*, which contained 64 GPCRs. GPCRs with amino-terminal extracellular cysteine-rich EGF-like domains (CFEM domain) are required for pathogenicity. In *M. grisea*, GPCRs with CFEM domain have been reported^37^. We found that no genes encoding GPCRs with CFEM domains were present in the genome of *T. horrida*. GPCRs suggest that *T. horrida* is a biotrophic fungi, which has adapted to a narrow host infection, and may acquire the ability to respond to a variety of environmental cues using different GPCRs that activate conserved intracellular signaling pathways and give new triggers to response systems. In particular, *T. horrida* contains more GPR124, thyrotropin-releasing hormone and secretagogue genes than other fungi. The identification and characterization of GPCRs will provide insights into the means by which *T. horrida* communicates with its environment and senses the presence of intracellular signaling.

Moreover, we predicted 24 homologues in the MAPK pathway. Amongst them, Ste3 pheromone receptors (*smut_6864* and *smut_6863*) were predicted, and components of other MAPK pathways were also detected, including Ste20 (*smut_7706*, *smut_7489*), Ste11 (*smut_5984*), Ste7 (*smut_4602*) and Ste12 (*smut_5905*). Of these Ste12 controls fungal virulence downstream of the pathogenic MAPK cascade as a master regulator of invasive growth in plant pathogenic fungi^38^. Determining the function of proteins such as Ste12 in the signal transduction pathway during host infection could elucidate the mechanisms by which fungal pathogens cause disease in plants.

## Discussion

*T. horrida* has developed into a major pathogen that restricts hybrid rice seed production. Here, we sequenced and assembled the draft genome of the highly virulent *T. horrida* strain JY-521. *T. horrida* has a genome size of 23.2 Mb, which is larger than that of other genus smut fungal genomes that vary from 18.38–21.15 Mb in size (Table 1). The genome contained 8.3% repeat sequences, most of which were TEs. However, compared with the genome size of the sequenced but not annotated *T. horrida* strain QB-1, using the NGS method, *T. horrida* JY-521 had a larger genome size^6^. Importantly, the *T. horrida* JY-521 genome was assembled used SMRT, which generates 1.9 Mb repetitive sequences that have not been identified in the genome of *T. horrida* QB-1 using other sequencing techniques^6^.

The sequence of *T. horrida* will provide useful information on host adaptation and evolutionary relationships in the smut species. A comparison of *T. horrida* with several smut fungi and some other important plant pathogens showed that smut fungi of the *Tilletia* genus and *Sporisorium*, and *Ustilago* genera were from different evolutionary branches, this suggests that species diversity of smut fungi and mechanisms of pathogen-host interaction differ in fungi from different smut. Benevenuto *et al*.^39^ compared the genomics of ten smut fungi isolated from maize, barley, sugarcane, wheat, oats, *Zizania latifolia* (Manchurian rice), *Echinochloa colona* (a wild grass), *Persicaria* sp. (a wild dicot plant), oats, and wheat; they showed that host domestication did not play a dominant role in shaping the evolution of smuts; however, the diversification and gain or loss of effector genes are probably the most important determinants of host specificity.

We compared the genomes of 14 fungi and demonstrated that biotrophic smut species contained many fewer genes than necrotrophic *Rhizoctonia solani* IA, the pathogen of rice sheath blight^31^. We also compared the number of GH families in biotrophic and hemi-biotrophic fungi. Results indicated that GH6, GH10, GH74, GH39, and GH43, that encode the enzymes of cellulose, hemi-cellulose, and pectin in *T. horrida*, were smaller than in *M. grisea*. These results showed that biotrophic *T. horrida* may minimize the degradation of host cell walls using carbohydrate-active enzymes, whose products are often recognized as endogenous signals to induce plant immunity^29,40^. These results also reflect the infection pattern of *T. horrida* that infects rice stamen filaments where cellulose and pectin are deficient^41^.

The effectors could interference with host immune responses to enhance virulence. For example, *Pseudomonas syringae* delivers over 30 effectors during infection. In order to clarify the function of fungal effectors, *N. benthamiana* has been extensively studied in attempts to elucidate the function of fungal effectors using agroinfiltration^42,43^. Several effectors that induce nonhost cell death were identified in *M. oryzae* and used in transient expression assays in *N. benthamiana* using agroinfiltration^44^. Similar studies have been reported in *P. sojae*^45^. We experimentally demonstrated that two selected putative effectors triggered cell death phenotypes in *N. benthamiana* (Fig. 8a) and they were regulated during *T. horrida* infection of rice panicles (Fig. 8c). The results showed that the two identified effectors may play important roles during the interaction of *T. horrida* and rice. Candidate effector proteins are highly diverse between smut species despite their close evolutionary relationship (Supplementary Dataset 2). These analyses further proved that adaptation to different hosts in smut species may be the result of divergent effector repertoires.

One set of characteristics often associated with effectors is a small size and high content of cysteine residues^46^. The secreted protein genes *smut_2965* and *smut_5844* were encoded 126 aa and 207 aa, respectively. In addition, plant pathogen effector genes can induce expression during infection^46^. This transcription data and quantitative real time reverse transcription-polymerase chain reaction (qRT-PCR) indicated that these two genes were induced during the early infection stage (Supplementary Fig. S13). Furthermore, through transient expression, we demonstrated that *smut_2965* and *smut_5844* could induce necrosis phenotypes in *N. benthamiana*. These results showed that *smut_2965* and *smut_5844* were effector genes and that they play an important role during *T. horrida*-rice interaction.

Over all, we reported a complete genome sequence of *T. horrida* using the SMRT sequencing method, which helped in the identification of repetitive elements. From genome assembly and annotation, we predicted that specific CAZymes, PHI genes, secondary metabolites, GPCR genes, and effectors can successfully help *T. horrida* to adapt to rice. Our study has also laid the groundwork for future discoveries on this important rice disease.

## Materials and Methods

### Strain isolates, culture conditions, and genomic DNA and RNA isolation

The RKS samples used for sequencing were collected from different heavily infected rice cultivars grown in different provinces of China. The *T. horrida* strains JY-521, CN-079, JS-058, GZ-102, SN-92, XJ-121, and HN-145 were separated using the spore suspension method^47^. All seven strains are haploid and were identified based on their mycelial morphology, using nucleus fluorescent staining and analysis of rDNA-ITS (internal transcribed spacer) sequences. The *T. horrida* strains were transferred into potato sugar agar (PSA) medium and incubated in the dark, with agitation at 200 rpm, at 30 °C for 5 d. Total DNA from fungal hyphae was extracted using the cetyltrimethylammonium bromide (CTAB) method^48^. The ITS rDNA genes were amplified using ITS primers (ITS-F:5′-TCCGTAGGTGAACCTGCGG-3′, ITS-R: 5′-TCCTCCGCTTATTGATATGC-3′). The sequencing of PCR products was undertaken by Sangon Biotech (Shanghai, China). Sequences were Blast-searched against NCBI databases. The nucleus fluorescent staining of *T. horrida* was performed according to the method of Hamada *et al*.^49^, and mycelial morphology was used as the standard for the detection and identification of *T. horrida* Tak^50^. The DNA of strain JY-521 was genome sequenced at Novogene Bioinformatics Technology (Beijing, China).

The strain JY-521 was used to infect rice cultivar 9311 A (which is highly susceptible to RKS) to create the treatment group. Young panicles of field grown rice plants, at the booting stage 3–5 d before heading, were collected during the late afternoon. The young panicles were disinfected with 75% alcohol for 2 min and washed twice with sterile water, and then air-dried for 20 min. Part of the hull of the panicles was cut off to allow the inoculation of individual colonies of *T. horrida* on PSA. Mycelium of *T. horrida* from six time points post-inoculation (0, 8, 12, 24, 48, and 72 h) were collected, immediately frozen in liquid nitrogen, and stored at −80 °C. Total RNA of *T. horrida* was isolated using the Omega Fungal RNA kit method. Dried RNA samples were dissolved in DEPC water. RNA quality was assessed on 1.0% denaturing agarose gels. Total RNA of *T. horrida* following infection of rice for 0, 8, 12, 24, 48, and 72 h was used for RNA-seq analysis.

### Genome sequencing and assembly

The genome of *T. horrida* strain JY-521 was sequenced using the Single Molecule Real-Time (SMRT) method at Novogene Bioinformatics Technology (Beijing, China). From the DNA, 20 kb insert PacBio RS II DNA sequencing libraries were constructed and three SMRT cells of raw data were generated (Supplementary Table S1). The output was 409,787 reads with an average length of 8,936 bp, resulting in 3,662,098,386 bp. The reads were corrected using the MinHash Alignment Process (MHAP)^51^, and the corrected reads were assembled using SOAP denovo^52,53^ and Celera Assembler^54^. DNA libraries with 300 bp inserts were constructed for the six other *T. horrida* strains. These DNA libraries were 150 bp paired-end sequenced using the Illumina HiSeq. 2000 at the Beijing Genomics Institute (BGI) in Shenzhen, China. Data from the Illumina libraries were first trimmed by removing low quality sequences and adapter sequences.

### Analysis of repeats

*T. horrida* repeat sequences were predicted by de novo and homology-based methods. De novo transposon libraries were constructed with the de novo software Piler v1.0^55^ and RepeatModeler (http://repeatmasker.org/RepeatModeler/, version 1.0.7). Transposable elements (TEs) were analyzed using libraries of the de novo method constructed using RepeatMasker (http://repeatmasker.org, version open-4.0.5). Tandem repeats sequences were predicted by Tandem Repeats Finder (TRF)^56^. All the parameters were set as default.

### Gene prediction and annotation

To obtain accurate candidate genes, we used three ab initio predictors: SNAP, GeneMark (Version 4.30), and AUGUSTUS to predict protein-coding genes in *T. horrida*^57–59^. The translation start sites were predicted using NetStart^60^. The six sets of RNA-seq raw data were aligned to the *T. horrida* JY-521 genome via TopHat v1.1.4 (ref.^50^), and exon junctions were predicted by aligning with the six RNA-seq libraries. Results of gene prediction were integrated using EuGene v3.6 (ref.^11^). The function of protein-encoding genes was annotated by BLASTp searches in the SwissProt (2015-07-24), KOG(2015-07-24), KEGG (2015-09-26), and NR (2015-07-24) databases, at the threshold of e-value ≤ 1e-5. GO annotation was accomplished by Blast2GO Pipeline (Version 2.3.5) with NR annotated results and the GO (2015-04-07) database.

### SNP identification

The high quality paired-ends read of the other six *T. horrida* strains were mapped to the assembled genome of *T. horrida* strain JY-521 using BWA software (version 0.7.12) with the command “mem –k 32 -M”, and BAM alignment files were generated using SAM tools software (version 0.1.19). The SNPs were identified using GATK software (version 3.4-46) with the Unified Genotyper model and were filtered using the Variant Filtration model and options -Window 4, -filter “QD < 4.0 || FS > 60.0 || MQ < 40.0 “, -G_filter “GQ < 20”. The filtered SNPs were annotated using ANNOVAR software (version 2014-07-14).

### Comparative genome analysis

The CDSs of gene proteins and CDSs sequences of from the 14 fungal genomes were collected, and compared using BlastP 2.6.0+ with the evalue ≤ 1e-7^61^. We removed short, spurious, and nonhomologous hits by setting a bitscore/alignment length filtering threshold of 0.4 and a minimum protein length of 30. Proteins passing this filter were clustered into families using the Markov clustering algorithm implemented by OrthoMCL software v1.4^62,63^ with options “–mode 3” -I as 2.0, and 806 single-copy gene families were obtained. The protein sequence of each single-copy family was aligned using MUSCLE v3.8.31, then, in turn, the protein alignment sequence to CDS alignment sequence and individual CDS alignment were concatenated into a string of nucleotide acids for each fugal genome. Regions that contained gaps or were highly divergent were removed from the data set using GBLOCKS software v0.91b^64^ with default parameters. Finally, the phylogenetic tree was built with MEGA v7.0.26^65^ under a neighbor-joining model and 1000 bootstrap replicates.

### Transcriptome expression

Total RNA was isolated from the *T. horrida* that infected rice panicles after 0, 8, 12, 24, 48, and 72 h using an RNA isolation kit according to the manufacturer’s instructions (Aidlab Biotechnologies, Beijing). These RNA were used for cDNA libraries constructed, and each library sequence was generated using a library with a read insert of 280 bp using IlluminaHiseq™ 2500 technology. We used mRNA-Seq for expression analysis. The RNA expression analysis was based on the predicted genes of *T. horrida*. A comparison map of mRNA reads in relation to the genome was generated by Tophat v2.0.14^66^, and the number of expected fragments of 1 kb of transcript per million fragments sequenced (FPKM) was calculated using Cufflinks^67^. The different expression genes were identified using EdgeR with FDR < 0.05 and |log2FC| > = 1^68,69^.

### Gene relation to pathogenicity and virulence-associated signaling pathway

For genes encoding proteins of carbohydrate metabolism, we used the Carbohydrate Active Enzymes (CAZy) database with an e-value of less than 1 × e^−5^. (2015-10-20) (http://www.cazy.org/). Secondary metabolism genes were predicted by SMURF (http://jcvi.org/smurf/precomputed.php) and NRPS predictor (version 2). Cytochrome P450 genes were identified in the P450 database with an e-value of ≤ 1 × e^−5^ (http://drnelson.uthsc.edu/cytochromeP450.html). The PHI-base database (http://www.phibase.org/) included experimentally verified disease-related genes of fungal and bacterial pathogens. So, we searched the *T. horrida* genome using the pathogen-host interaction database with an e-value of ≤ 1 × e^−5^, and pathogen-host interaction genes were identified. ABC transporters in the UniProt database (http://www.uniprot.org/) were identified using BLAST with an e-value of 8 × e^−14^ and the TCDB with an e-value ≤ 1 × e^−5^.

Important elements of the G-protein pathway were identified using BLAST with a threshold e-value of ≤ 1 × e^−5^ (http://www.genome.jp/kegg/pathway/sce/sce04011.html). The identification and verification of seven transmembrane (7-TM) helices of GPCR-like proteins was performed using TMPRED (http://www.ch.embnet.org/software/TMPRED_form.html), Phobius, and TMHMM 2.0c (http://phobius.sbc.su.se/, http://www.cbs.dtu.dk/services/TMHMM/). The GPCR-like proteins were classified using the following website: http://fse2013.spms.ntu.edu.sg/~chenxin/PCA_GPCR/.

### Secreted proteins

There are several prediction algorithms that may be used in the analysis of secreted proteins of *T. horrida*. The signal peptide site of potential secreted proteins was predicted by SignalP4.0 (http://www.cbs.dtu.dk/services/SignalP-4.0/) and Phobius (http://phobius.binf.ku.dk/). Transmembrane helices in the proteins were predicted using TMHMM 2.0 (http://www.cbs.dtu.dk/services/TMHMM-2.0/). Proteins located in the mitochondria, as determined by TargetP (http://www.cbs.dtu.dk/services/TargetP/), were removed. The proteins that contained signal peptide cleavage sites and no transmembrane helices were potential secreted proteins.

### Candidate effectors and their validation

Putative effectors in the *T. horrida* secretomes were predicted based on the protein size (≤400 amino-acid residues) and expression during the early infection stage of the rice-fungus interaction^70,71^. The functions of putative effector proteins were identified by expressed used transient expression assay in *N. benthamiana* by agroinfiltration. All DNA manipulations and other procedures, including agarose gel electrophoresis, were performed according to standard protocols.

### Expression vector construction and preparation

A total of 131 expression plasmids were constructed with the PMDC32 Expression Vector (Libo Shan, Texas A&M University). RNA was prepared from *T. horrida* mycelia using a Fungal RNA kit (Omega), and cDNA was synthesized using a Transcriptor First strand cDNA synthesis kit (Roche). All PCR products used for cloning were generated using Trans Start FastPfu Fly DNA Polymerase (TransGen Biotech, Beijing, China). All of the restriction enzymes and ClonExpress enzymes were used following the manufacturer’s instructions (Vazyme Biotech, Nanjing, China). Primers for these assays were designed based on our predicted gene sequences and included a BamHI site and a StuI site used CE Design v1.03. The obtained cDNA of target genes were gel-purified with a gel purification kit (Omega) and cloned into the PMDC32 Expression vector.

### Transient protein expression in N. benthamiana

For *N. benthamiana* leaf transformations, PMDC32 expression vector constructs were transformed into *Agrobacterium tumefaciens* strain GV3101. Bacterial strains were grown in YEP liquid medium containing 50 mg/mL rifampicin, and 50 mg/mL kanamycin at 28 °C for 16 h. Bacteria were harvested by centrifugation, resuspended in infiltration medium [10 mM MES (pH 5.6), 10 mM MgCl_2_, and 150 *μ*M acetosyringone] to an OD 600 at 0.5, and incubated in the dark for 3 h at room temperature before leaf infiltration. For each independent infiltration experiment, each construct was infiltrated on 20 leaves from different plants. The infiltrated plants were incubated in growth chambers under controlled conditions for all following assays. For documentation of cell death, leaves were photographed 4–7 d after infiltration.

### Plant growth conditions and infection assay conditions

*N. benthamiana* plants were housed in a growth chamber under light for 16 h at 23 °C and in the dark for 8 h at 23 °C. Spore suspension was inoculated onto plants at the 4-leaf stage using a syringe. The vector containing the BAX gene inoculant was used as the positive control, and a vector containing the green fluorescent protein (GFP) gene inoculation was used as the negative control. Similar results were obtained in five independent experiments. The leaf cell death phenotypes were observed 4d post infiltration.

### qRT-PCR

To further verify the expression trend of key genes, qRT-PCR was performed with a Bio-Rad CFX96 Real-Time PCR System (Foster City, CA, USA), according to the manufacturer’s instructions. The ubiquitin (UBQ) gene was used as an internal control for data normalization. The expression levels of genes were calculated using the 2^−∆∆Ct^ algorithm. Primers used for qRT-PCR are listed in Supplementary Table S16.

## Electronic supplementary material

supplementary figure and table

supplementary dataset 1

supplementary dataset 2

supplementary dataset 3

## Data Availability

The authors declare that all data of this study are available from the corresponding author upon reasonable request.
