# Supplementary material for: The pathogenic mechanisms of Tilletia horrida as revealed by comparative and functional genomics
Source: Sci Rep. 2018 Oct 18;8:15413. doi: 10.1038/s41598-018-33752-w (PMC6194002; doi:10.1038/s41598-018-33752-w)
Supplement: Supplementary file 1 — supplementary figure and table [file 41598_2018_33752_MOESM1_ESM.docx]

**The pathogenic mechanisms of *Tilletia horrida* as revealed by comparative and functional genomics**

Aijun Wang^1,2,3^, Linxiu Pang^1^, Na Wang^1^, Peng Ai^1^, Desuo Yin^4^, Shuangcheng Li^1,2,3^, Qiming Deng^1,2,3^, Jun Zhu^1,2,3^, Yueyang Liang^1,2,3^, Jianqing Zhu^1,2,3^, Ping Li^1,2,3^, Aiping Zheng^1,2,3^

^1^Rice Research Institute of Sichuan Agricultural University, Wenjiang, Chengdu, Sichuan, 611130, China

^2^Key laboratory of Sichuan Crop Major Disease, Sichuan Agricultural University, Wenjiang, Chengdu, Sichuan, 611130, China

^3^Key Laboratory of Southwest Crop Gene Resource and Genetic Improvement of Ministry of Education, Sichuan Agricultural University, Yaan, Sichuan, 611130, China

^4^Food Crop Research Institute, Hubei Academy of Agricultural Science, Wuhan, Hubei, 611130, China


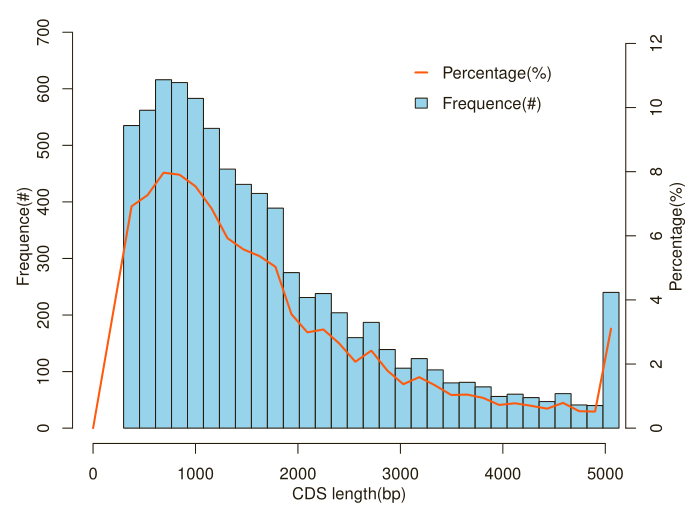


**Supplementary Figure S1** The CDS length distribution of *T. horrida.*


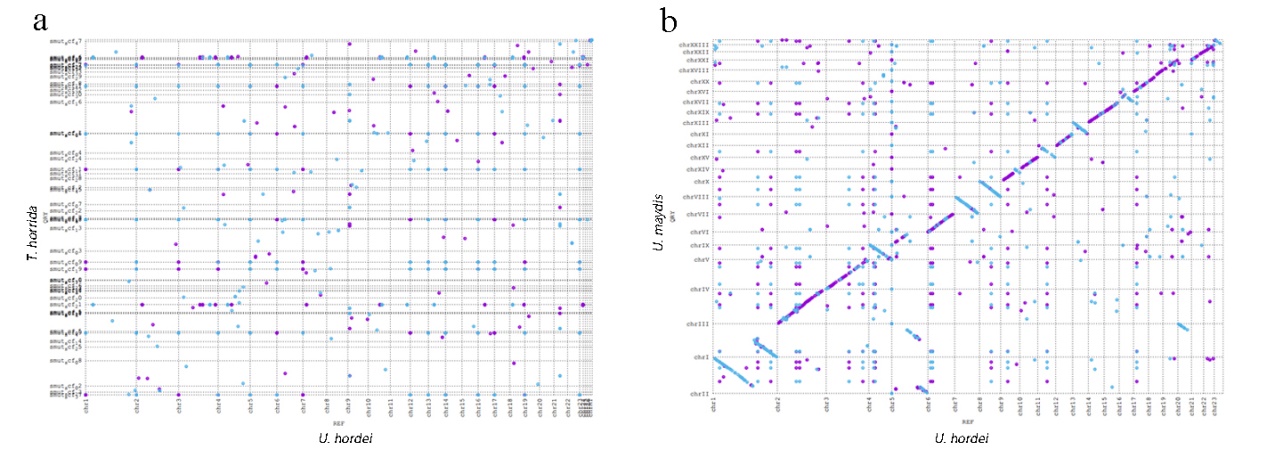


**Supplementary Figure S2** (a) Genome assembly sequence comparisons between *T. horrida* and *U. hordei*. (b) Genome assembly sequence comparisons between *U. maydis* and *U. hordei*. Dot-plots depict nucleotide sequence matches detected via MUMmer (nucmer) between the two seqences of *T. horrida* and *U. hordei*, as well as homologous from *U. hordei* and *U. maydis*.


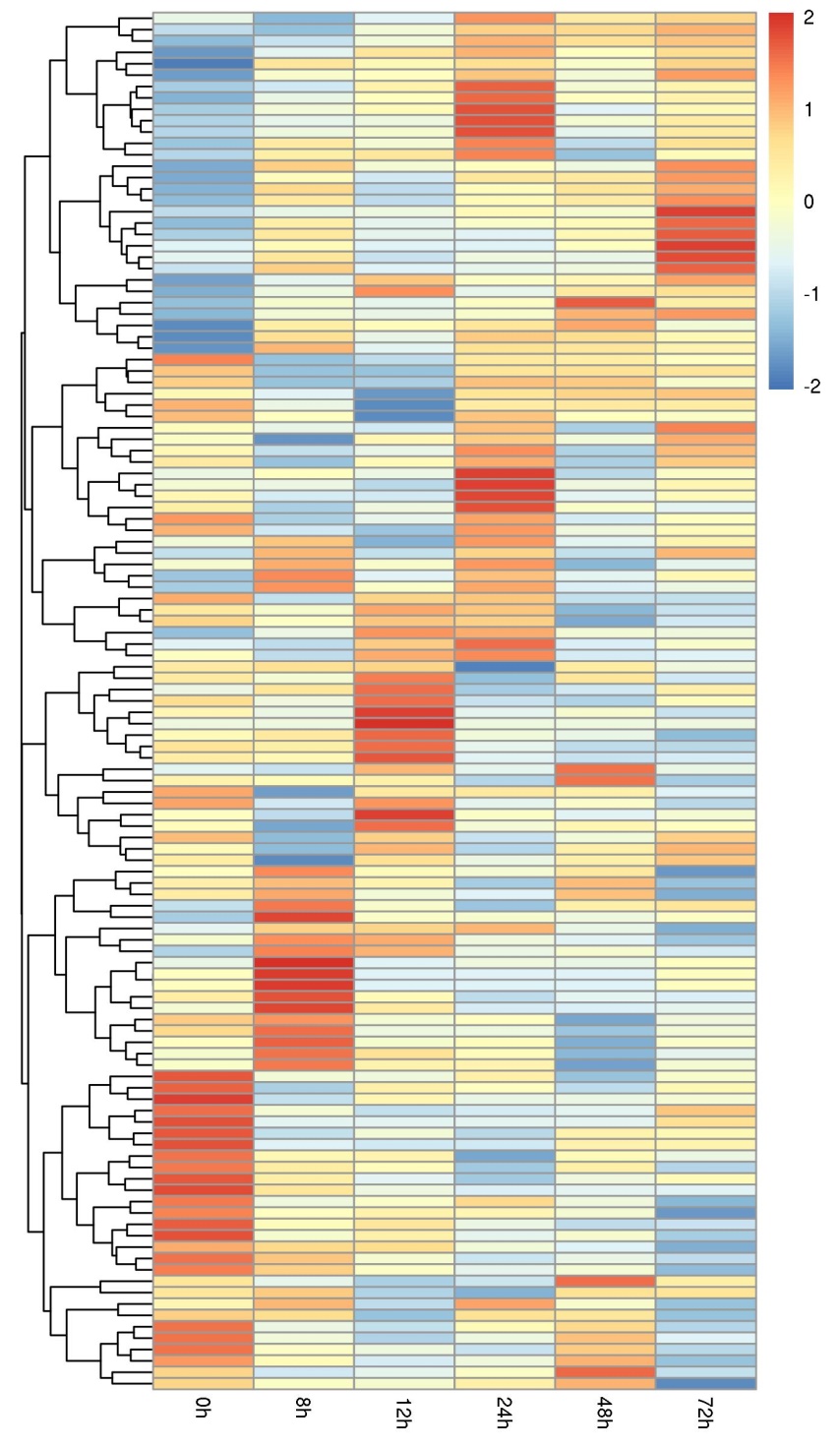


**Supplementary Figure S3** 138 unnamed or uncharacterized protein encoding genes expressed in *T. horrida* during the host infection.


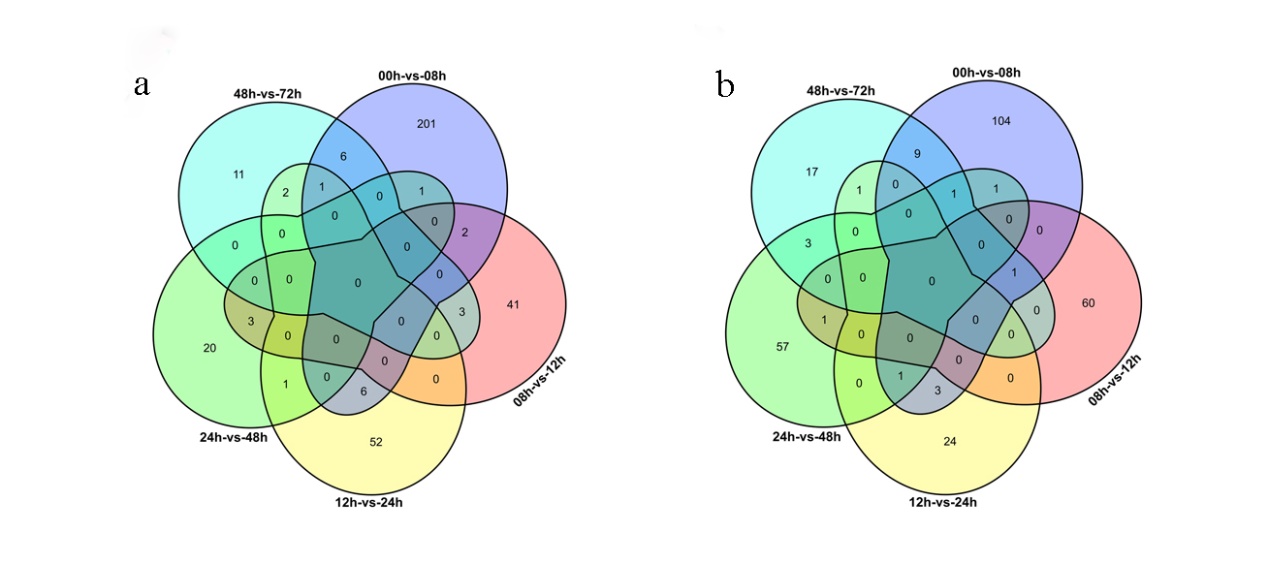


**Supplementary Figure S4** The significant down-regulated and significant up-regulated genes after infection statistics.


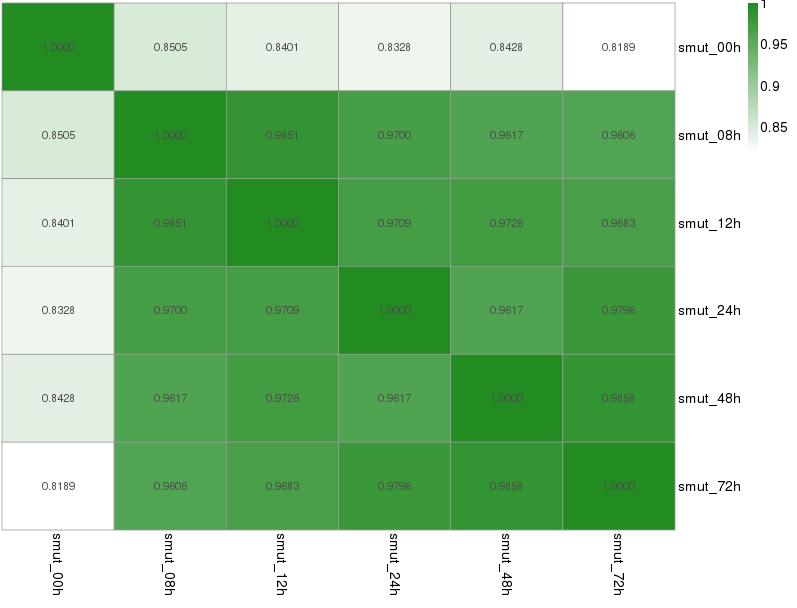


**Supplementary Figure S5** Sample lustering of different infection times.


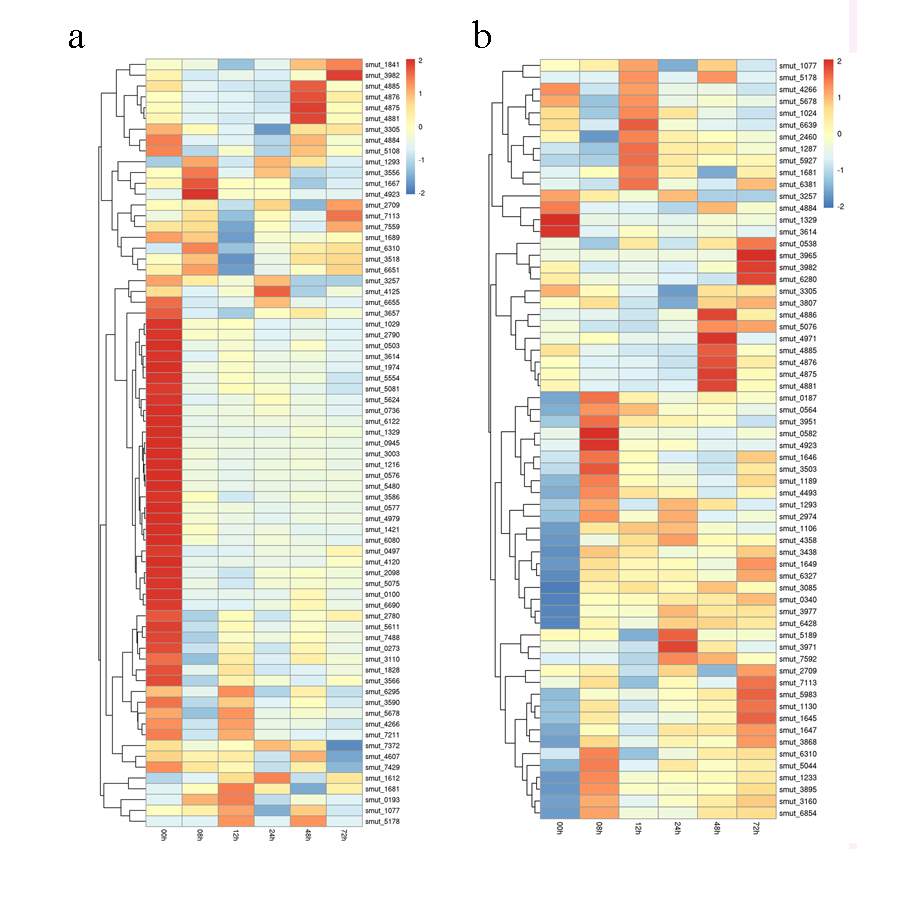


**Supplementary Figure S6** PHI genes expressed in *T. horrida* during the host infection. (a) The significant up-regulated PHI gene after infected. (b) The significant down-regulated PHI gene after infected.


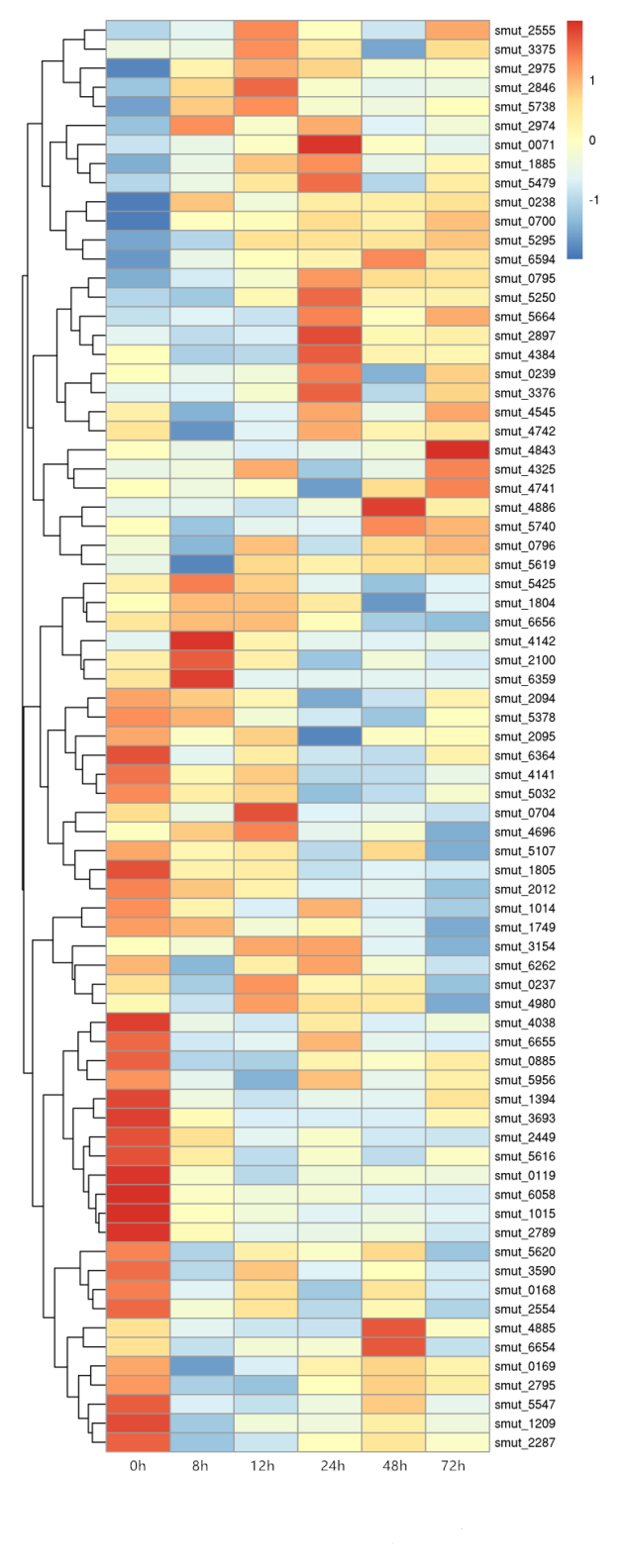


**Supplementary Figure S7** Secondary metabolite genes expressed in *T. horrida* during the host infection.


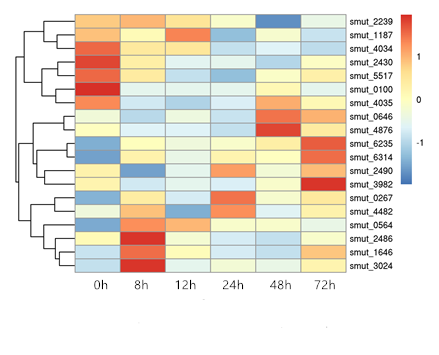


**Supplementary Figure S8** Cytochrome P450s family genes expressed in *T. horrida* during the host infection.

**
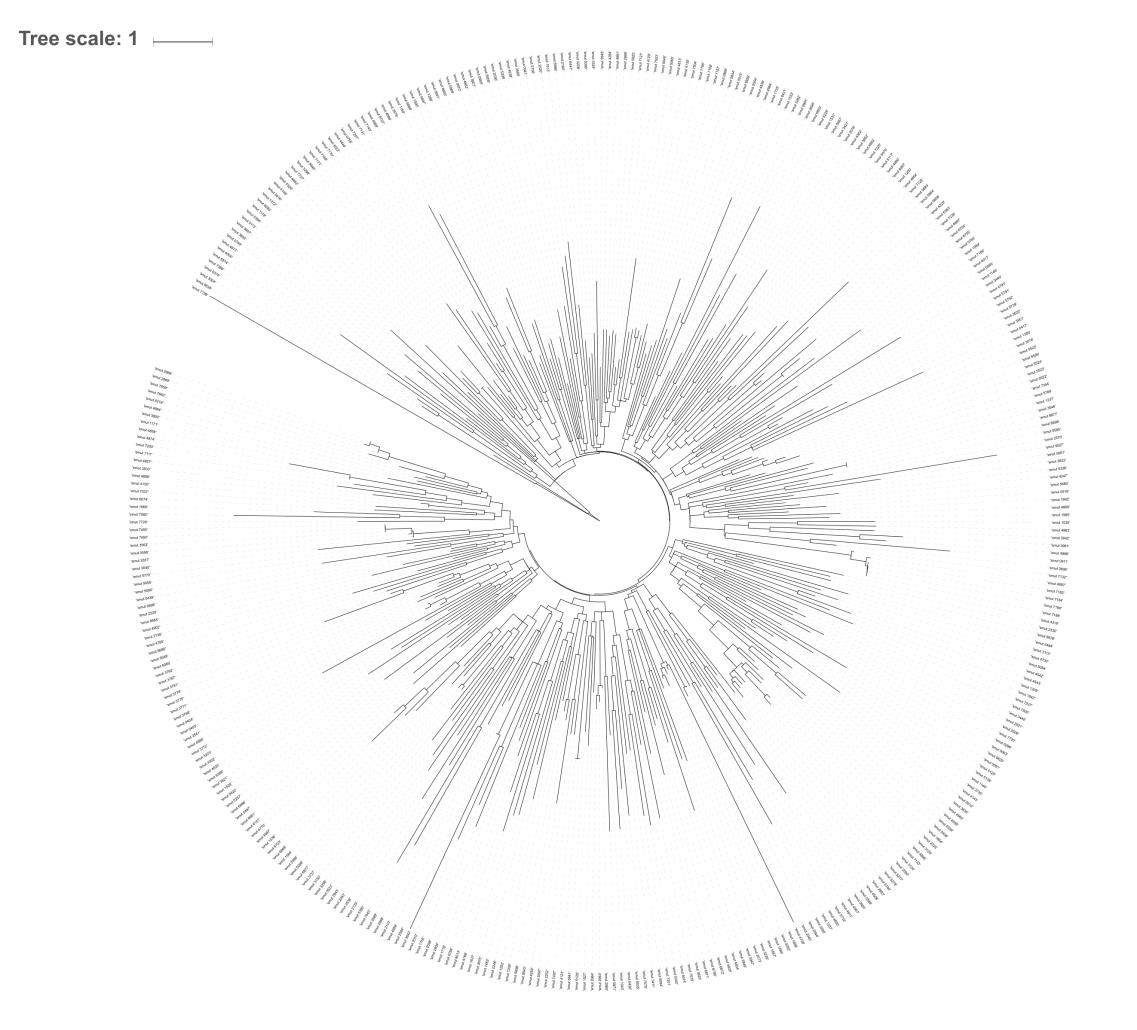
**

**Supplementary Figure S9** A phylogenetic trees of 366 putative effectors indicates that most effectors are categorized into several super clades.


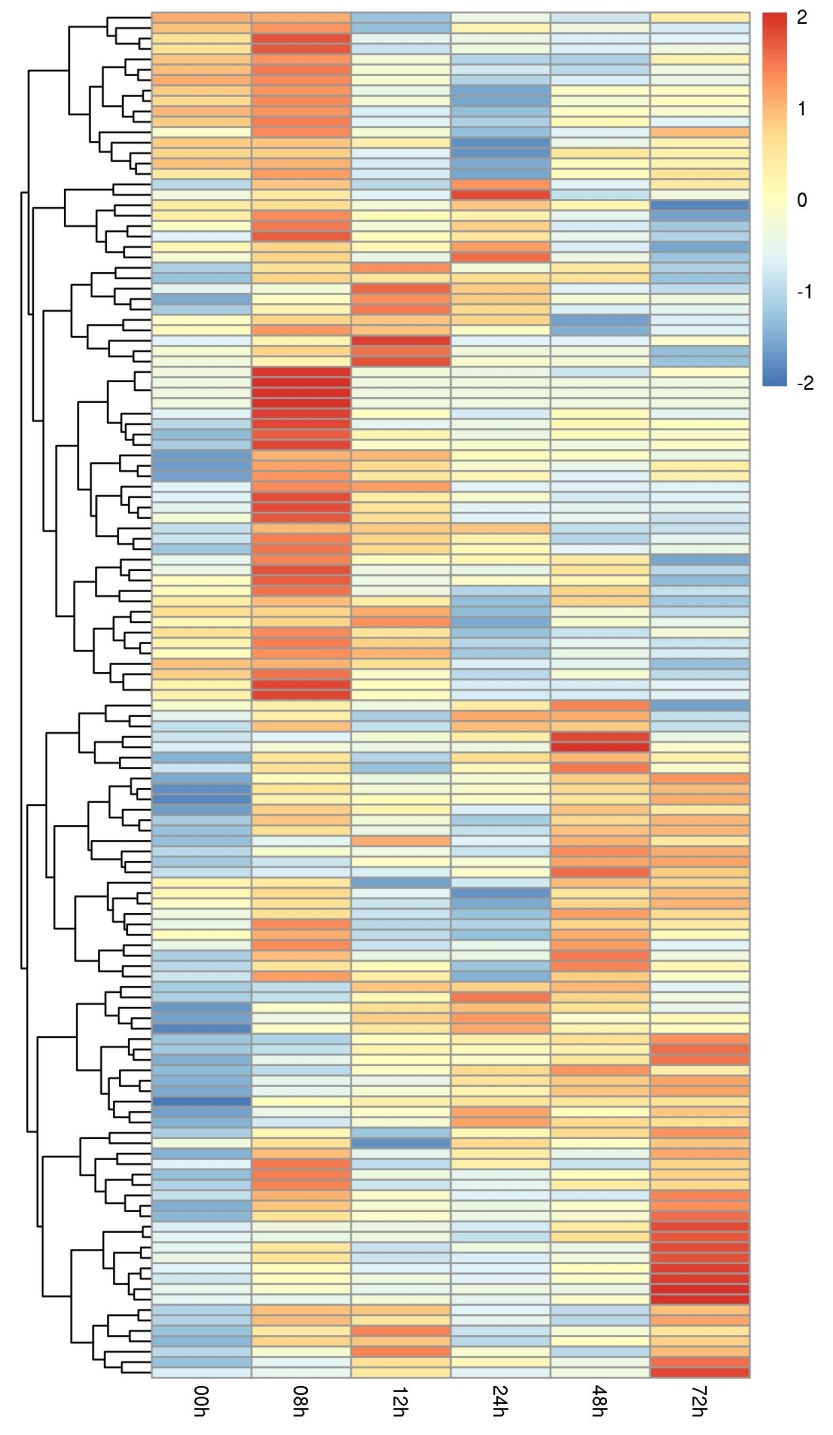


**Supplementary Figure S10** Secretome genes expressed in *T. horrida* during the host infection.

**
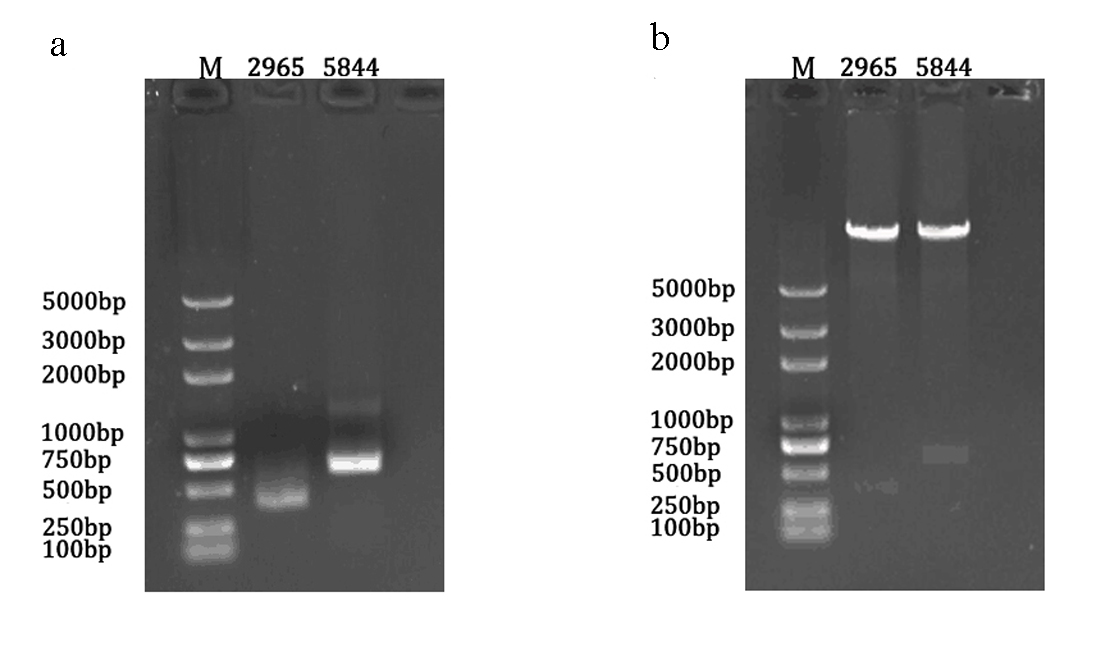
**

**Supplementary Figure S11** (a) The genes of Smut_2965 and Smut_5844 PCR; (b) When the plasmids are digested by BamHI and StuⅠ.


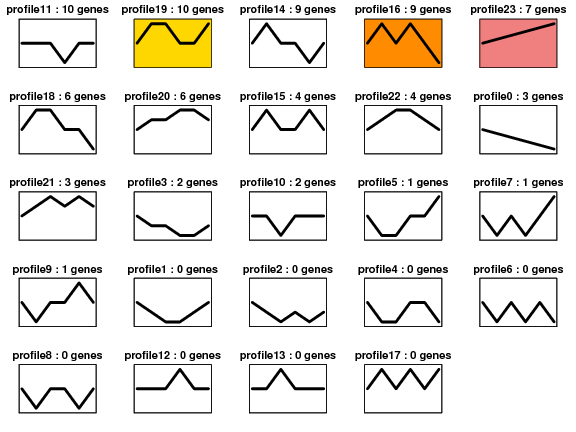


**Supplementary Figure S12** The trend analysis of putative effectors proteins.


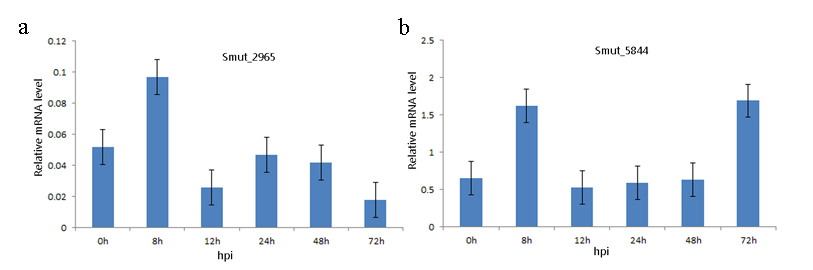


**Supplementary Figure S13** Expression profiles of smut_5844 and smut_2965 during *T. horrida* infection of the rice male sterile line 9311A. Mycelium of *T. horrida* from six time points post-inoculation (0, 8, 12, 24, 48, and 72 h) were collected for gene expression analyses using quantitative real time reverse transcription-polymerase chain reaction assay. Data are means ± standard error. Gene expression patterns shown are representatives from five independent repeats with similar results.

**Supplementary Table S1 Summary of the high-quality PacBio RS II sequenced data**

| cells | Polymerase read Bases(bp) | Number of Polymerase reads | Mean Read Length | Read N50 Polymerase | Polymerase Read Quality | Mean Subread length(bp) | subreads N50 | Number of subreads(bp) |
| --- | --- | --- | --- | --- | --- | --- | --- | --- |
| D11_1 | 1,169,523,583 | 82,043 | 14,255 | 21,105 | 0.85 | 7,478 | 10,180 | 140,504 |
| E08_1 | 1,241,723,680 | 95,109 | 13,055 | 18,597 | 0.84 | 8,776 | 12,150 | 141,230 |
| F08-1 | 1,257,278,943 | 98,137 | 12,811 | 18,614 | 0.84 | 8,800 | 12,206 | 142,624 |
| Total | 3,668,526,206 | 275,289 | 13,326 | - | 0.84 | 8,354 | - | 424,358 |

**Supplementary Table S2 Description of *T. horrida* genome assembly**

| Statistics | Scaffold | Contig |
| --- | --- | --- |
| Total number(#) | 84 | 84 |
| Total length(bp) | 23,215,372 | 23,215,372 |
| Gap(N)(bp) | 0 | 0 |
| Average length(bp) | 276,373.48 | 276,373,48 |
| N50 length(bp) | 538,348 | 538,348 |
| GC content | 55.67% | 55.67% |

**Supplementary Table S3 Protein database annotation result for protein of *T. horrida***

| **Database** | **Number** | **(%)** |
| --- | --- | --- |
| SwissProt | 4,959 | 64.16 |
| GO | 6,462 | 83.61 |
| KEGG | 5,540 | 71.69 |
| KOG | 4,324 | 55.95 |
| NR | 6,212 | 80.37 |
| Common | 4,220 | 54.6 |

**Supplementary Table S4 Noncoding genes in the *T. horrida* genome**

| Type | | Copy Number | Average Length (bp) | Total Length (bp) |
| --- | --- | --- | --- | --- |
| tRNA | | 155 | 80 | 12,410 |
| rRNA | 18S | 14 | 1,773 | 24,815 |
|  | 28S | 14 | 5,364 | 75,100 |
|  | 8S | 13 | 112 | 1,462 |

**Supplementary Table S5 Summary of transposable elements of *T. horrida***

| class | family | count | Length(bp) | Percentage(%) |
| --- | --- | --- | --- | --- |
| DNA | *MuLE-MuDR* | 508 | 8,804 | 0.038 |
|  | *PIF-Harbinger* | 41 | 59,046 | 0.254 |
|  | *Tcmar-Tc1* | 546 | 38,981 | 0.168 |
|  | *hAT-Ac* | 98 | 2,486 | 0.011 |
| LINE | *Penelope* | 8 | 51,175 | 0.22 |
|  | *R2-Dualen* | 27 | 7,893 | 0.034 |
| LTR | *Copia* | 1,956 | 230,297 | 0.992 |
|  | *Gypsy* | 1,408 | 835,814 | 3.6 |
|  | *Ngaro* | 160 | 2,851 | 0.012 |
|  | *Pao* | 141 | 2,578 | 0.011 |
| Simple repeat | | 175 | 28,664 | 0.123 |
| Unknow | | 9,463 | 692,178 | 2.982 |
| Total | | 14,531 | 1,958,189 | 8.445 |

Abbreviations: LINE, long interspersed elements; LTR, long terminal repeat retrotransposons

**Supplementary Table S6 General information for the six other *T. horrida* strains sequenced in this study**

| Strains | Places collected | Host cultivars | Year collected | Insert size (bp) | Reads length (bp) | Clean date (Mb) |
| --- | --- | --- | --- | --- | --- | --- |
| JY-521 | Anhui Province in China | XianJingA | 2016 |  |  |  |
| CN-079 | Wenjiang, Sichuang Province in China | JinLongA | 2016 | 300 | 150 | 1,339 |
| JS-058 | Jiangsu Province in China | FanYuanA | 2016 | 300 | 150 | 1,344 |
| GZ-102 | Guizhou Province in China | FuNongA | 2016 | 300 | 150 | 1,339 |
| SN-92 | Suining, Sichuang Province in China | Gan73A | 2016 | 300 | 150 | 1,343 |
| XJ-121 | Xinjing, Sichuang Province in China | DShanA | 2016 | 300 | 150 | 1,344 |
| HN-145 | Hunan Province in China | Feng39S | 2016 | 300 | 150 | 1,335 |

**Supplementary Table S7 Summary of SNPs in different *T. horrida* strains when compared with the reference strain JY-521**

| Strain | SNP number | Unique SNP | SNPs in intergenic | SNPs in gene region | SNPs in introns | SNPs in exons | Total genes with SNPs | Effectors with SNPs | PHI genes with SNPs |
| --- | --- | --- | --- | --- | --- | --- | --- | --- | --- |
| CN-079 | 99,796 | 19,663 | 31,741 | 68,055 | 13,621 | 54,434 | 6,312 | 299 | 1,451 |
| JS-058 | 103,715 | 19,844 | 32,788 | 70,927 | 14,329 | 56,598 | 6,519 | 296 | 1,513 |
| GZ-102 | 69,953 | 12,519 | 21,953 | 48,000 | 9,354 | 38,646 | 4,761 | 225 | 1,118 |
| SN-92 | 98,986 | 19,912 | 31,678 | 67,308 | 13,489 | 53,819 | 6,264 | 301 | 1,452 |
| XJ-121 | 118,995 | 24,832 | 38,340 | 80,655 | 16,122 | 64,533 | 6,615 | 311 | 1,517 |
| HN-145 | 124,367 | 21,601 | 39,808 | 84,559 | 17,134 | 67,425 | 6,760 | 316 | 1,541 |

**Supplementary Table S8 Summary of gene family clustering of *T. horrida***

| Species | Genes number | Families number | Unique families | Unique genes | Single-gene family | Genes per family |
| --- | --- | --- | --- | --- | --- | --- |
| *L. bicolor* | 23127 | 19495 | 12816 | 14386 | 12079 | 1.19 |
| *M. grisea* | 11054 | 10287 | 3959 | 4044 | 3896 | 1.07 |
| *M.lini* | 16335 | 13873 | 7229 | 7823 | 6812 | 1.18 |
| *M.laricis-populina* | 19550 | 16625 | 9266 | 9767 | 8907 | 1.18 |
| *P. graminis* | 15979 | 13334 | 6267 | 6650 | 6021 | 1.2 |
| *P. striformis* | 20482 | 16252 | 8975 | 9532 | 8613 | 1.26 |
| *R.solani* | 10489 | 9852 | 5287 | 5313 | 5262 | 1.06 |
| *S. commune* | 16293 | 13905 | 7059 | 7605 | 6724 | 1.17 |
| *S. reilianum* | 6648 | 6469 | 202 | 205 | 201 | 1.03 |
| *S. scitamineum* | 6616 | 6321 | 336 | 362 | 322 | 1.05 |
| *T. caries* | 10204 | 9018 | 1069 | 1075 | 1065 | 1.13 |
| *T. controversa* | 9860 | 8691 | 801 | 807 | 795 | 1.13 |
| *T. horrida* | 7729 | 6662 | 1166 | 1260 | 1102 | 1.16 |
| *T. indica* | 9548 | 8554 | 1606 | 1636 | 1579 | 1.12 |
| *T. walkeri* | 7970 | 7387 | 675 | 676 | 674 | 1.08 |
| *U. hordei* | 7110 | 6847 | 871 | 882 | 863 | 1.04 |
| *U. maydis* | 6783 | 6528 | 387 | 396 | 381 | 1.04 |
| *U. virens* | 8426 | 8123 | 2432 | 2439 | 2425 | 1.04 |

**Supplementary Table S9 Comparative analysis of CAZyme family among various plant pathogen fungi**

| Species | CBM | CE | GH | GT | PL | Total |
| --- | --- | --- | --- | --- | --- | --- |
| *U. maydis* | 252 | 85 | 449 | 374 | 18 | 1178 |
| *U. hordei* | 249 | 70 | 573 | 516 | 44 | 1452 |
| *S. scitamineum* | 251 | 76 | 430 | 356 | 15 | 1128 |
| *T. horrida* | 273 | 101 | 474 | 533 | 43 | 1424 |
| *S. reilianum* | 258 | 80 | 436 | 344 | 15 | 1133 |
| *P. striformis* | 400 | 101 | 587 | 506 | 26 | 1620 |
| *S. commune* | 535 | 156 | 841 | 581 | 42 | 2155 |
| *M.lini* | 338 | 92 | 524 | 439 | 25 | 1418 |
| *M.laricis-populina* | 399 | 142 | 584 | 418 | 28 | 1571 |
| *P. graminis* | 371 | 89 | 540 | 422 | 20 | 1442 |
| *R.solani* | 408 | 92 | 546 | 386 | 54 | 1486 |
| *L. bicolor* | 551 | 125 | 801 | 702 | 48 | 2227 |
| *M.grisea* | 499 | 186 | 841 | 534 | 39 | 2099 |
| *U.virens* | 293 | 79 | 517 | 406 | 20 | 1315 |

**Supplementary Table S10 The different counts of up-regulated CAZymes involved in the five infection stages**

| CAZy | 8h | 12h | 24h | 48h | 72h |
| --- | --- | --- | --- | --- | --- |
| CBM | 147 | 214 | 130 | 120 | 30 |
| CE | 46 | 57 | 31 | 52 | 21 |
| GH | 204 | 315 | 189 | 194 | 56 |
| GT | 242 | 309 | 180 | 211 | 72 |
| PL | 13 | 17 | 12 | 17 | 6 |
| TOTAL | 652 | 912 | 542 | 594 | 185 |

**Supplementary Table S11 The different quantities of up-regulated cellulose, hemi-cellulose and pectin- degrading enzymes involved in the five infection stages**

| CAZymes |  | 8h | 12h | 24h | 48h | 72h |
| --- | --- | --- | --- | --- | --- | --- |
| Cellulose | GH1 | 2 | 2 | 0 | 2 | 1 |
|  | GH3 | 5 | 5 | 1 | 7 | 3 |
|  | GH5 | 11 | 17 | 10 | 12 | 8 |
|  | GH45 | 4 | 3 | 2 | 3 | 1 |
|  | GH12 | 1 | 1 | 0 | 0 | 0 |
| Hemicellulose Degrading enzymes | GH10 | 0 | 1 | 0 | 0 | 0 |
|  | GH16 | 20 | 21 | 14 | 17 | 9 |
|  | GH43 | 7 | 16 | 7 | 10 | 1 |
|  | GH27 | 0 | 0 | 2 | 2 | 0 |
|  | GH29 | 1 | 0 | 1 | 1 | 0 |
|  | GH39 | 1 | 1 | 0 | 0 | 0 |
|  | GH31 | 2 | 4 | 2 | 2 | 1 |
|  | GH35 | 1 | 2 | 1 | 0 | 0 |
|  | GH36 | 12 | 12 | 7 | 6 | 2 |
| Pectin Degrading enzymes | CE8 | 3 | 6 | 6 | 5 | 2 |
|  | PL4 | 0 | 2 | 1 | 2 | 0 |
|  | PL1 | 2 | 7 | 4 | 7 | 1 |
|  | GH88 | 0 | 1 | 0 | 1 | 0 |
|  | GH78 | 4 | 7 | 3 | 8 | 0 |
|  | GH28 | 12 | 7 | 9 | 10 | 5 |
|  | GH43 | 7 | 16 | 7 | 10 | 1 |
|  | GH105 | 1 | 1 | 0 | 2 | 0 |
| TOTAL |  | 96 | 132 | 77 | 107 | 35 |

**Supplementary Table 12 Prediction of the secondary metabolite biosynthesis gene clusters**

| Core gene id | Gene cluster type | software |
| --- | --- | --- |
| Smut_0071 | NRPS | SMURF |
| Smut_0795 | NRPS-Like | SMURF |
| Smut_1014 | NRPS-Like | SMURF |
| Smut_2974 | PKS | SMURF |
| Smut_4886 | Hybrid | SMURF |
| Smut_6655 | PKS-Like | SMURF |
| Smut_6656 | PKS | SMURF |

**Supplementary Table S13 Prediction of secreted proteins of *T. horrida***

| Species | Total secreted(per%) | gpi | Small cysteine-rich |  |
| --- | --- | --- | --- | --- |
| *U. hordei* | 652(9.17) | 31(0.44) | 40(0.56) | |
| *U. maydis* | 701(10.33) | 32 (0.49) | 36 (0.38) | |
| *S. scitamineum* | 718(9.31) | 23(0.41) | 31(0.46) | |
| *S. reilianum* | 543(8.17) | 27(0.39) | 43(0.65) | |
| *T. horrida* | 597(7.72) | 24(0.31) | 35(0.45) | |
| *R.solani* | 965(9.20) | 20(0.19) | 103(0.98) | |
| *U.virens* | 628(7.45) | 26(0.28) | 193(2.29) | |

**Supplementary Table S14 The 14 co-expression genes with smut_2965 and smut_5844**

| Gene_id | Type | NR description | evalue |
| --- | --- | --- | --- |
| Smut_0749 | secretion protein | — | — |
| Smut_1035 | secretion protein | uncharacterized protein UHOR_02471 | 1.79E-18 |
| Smut_1149 | secretion protein | — | — |
| Smut_2965 | secretion protein | guanyl-specific ribonuclease | 2.18E-37 |
| Smut_4856 | secretion protein | — | — |
| Smut_7720 | secretion protein | carotenoid ester lipase precursor | 1.86E-22 |
| Smut_0691 | secretion protein | endo-glucanase RCE3 | 4.56E-40 |
| Smut_1985 | secretion protein | — | — |
| Smut_3800 | secretion protein | — | — |
| Smut_4229 | secretion protein | — | — |
| Smut_5040 | secretion protein | zincin | 1.91E-52 |
| Smut_5065 | secretion protein | — | — |
| Smut_5125 | secretion protein | predicted protein | 4.37E-126 |
| Smut_5309 | secretion protein | — | — |
| Smut_5844 | secretion protein | — | — |
| Smut_6705 | secretion protein | — | — |

**Supplementary Table S15 Homologous gene of smut_2965**

| Species | *T. horrida* | *U. hordei* | *U. maydis* | *S. reilianum* | *S. scitamineum* |
| --- | --- | --- | --- | --- | --- |
| Homologous gene | Smut_2964, Smut_2963 | Uho_2533 | Uma_1899 | Sre_4650 | Ssc_1628 |
|  |  |  |  |  |  |

**Supplementary Table S16 The primer sequence of qRT-PCR**

| name | primer sequence |
| --- | --- |
| smut_5844F | ATGAAGCTTGCGATTGCAACC |
| smut_5844R | AGCGACGGTAGCGATCGTC |
| smut_2965F | ATGAAGTTTGCCACCCTCGC |
| smut_2965R | GTAAGAGCACTTAAGGAAGCTGCTG |
| UBQF | CCCATCAACATCATTGTCGCT |
| UBQR | TTCACACCCTTTCGCATCTG |
